# The impacts of neighbourhood violence on mental health consultations among residents of low-income neighbourhoods in Rio de Janeiro: a retrospective cohort analysis

**DOI:** 10.1186/s13033-026-00702-3

**Published:** 2026-04-10

**Authors:** Sophia Medeiros, Vinicius Peçanha, Julia Guerra, Gustavo Cordeiro, Aldina Mesic, Rudi Rocha, Christopher Millett, Thomas Hone

**Affiliations:** 1 Department of Primary Care and Public Health, Public Health Policy Evaluation Unit, School of Public Health, Imperial College London, White City Campus, London, W12 7TA UK; 2 Instituto de Estudos para Políticas de Saúde (IEPS), São Paulo, Brazil; 3 Escola de Administração de Empresas de São Paulo da Fundação Getulio Vargas (FGV EAESP), São Paulo, Brazil; 4 Public Health Research Center, Comprehensive Health Research Center, NOVA National School of Public Health, NOVA University Lisbon, Lisbon, Portugal

**Keywords:** Mental health, Violence, Rio de Janeiro, Brazil, LMIC, Urban health

## Abstract

**Background:**

Violence is associated with poor mental health outcomes. However, longitudinal evidence from urban settings with endemic violence – particularly in low- and middle-income countries such as Brazil – remains limited. This study investigates the association between neighbourhood violence, including drug-gang conflicts and police operations, on mental health consultations in Rio de Janeiro.

**Methods:**

A cohort of 1,358,943 individuals observed from January 2010 to December 2016 was analysed. Individuals were considered exposed to violence if a violent event occurred within 250 m of the primary healthcare clinic they attended. Using cross-sectional Poisson regression models, we examined which sociodemographic groups were more exposed to neighbourhood violence, and fixed effects panel Poisson regression assessed the association between neighbourhood violence and PHC consultations for mental health conditions.

**Results:**

Over two thirds of the cohort (*n* = 913,125) were exposed to at least one neighbourhood violent event in the study period, most of which were police-related shootings (70.4%). Females, Black individuals, and younger adults were more likely to experience neighbourhood violence, particularly drug-gang violence. Exposure to two and three neighbourhood violent events in a month was associated with a 5% (IRR: 1.05; 95% CI: 1.02–1.07; *p* < 0.001) and 8% (IRR: 1.08; 95% CI: 1.04–1.12; *p* < 0.001) increase in mental health-relatedprimary healthcare consultations, respectively, while five or more events were linked to a 7% decrease (IRR: 0.93; 95% CI: 0.89–0.97; *p* < 0.001). Lagged analyses showed that exposure to 2–4 violent events per month was associated with increased mental health consultations, particularly 2–4 months after violence exposure. Increases in mental health consultations following high levels of violence exposure were observed among individuals aged 19–34 and 65 + years, but were lower among those with a high school education or higher.

**Conclusions:**

Neighbourhood violence in Rio de Janeiro was associated with increased mental health-related primary healthcare consultations both immediately and two to four months following violence exposure.

## Background

Violence poses a significant challenge for global and human development. Each year, violence – including interpersonal, self-directed, and collective forms – kills an estimated 1.25 million people and causes tens of millions more non-fatal injuries and long-term health consequences [1, 2]. For people aged 15–44 years, violence is among the leading causes of death worldwide [2]. Violence, specifically interpersonal violence, remains a critical issue in Brazil, with the country’s homicide rate reaching 30.8 per 100,000 people in 2020 – three times the global average [3, 4]. Beyond the numerous deaths caused by violence, Brazil falls in the top 20% of countries with the highest disability adjusted life years per 100,000 population due to interpersonal violence [5]. 

Being victim to, or witnessing violence increases the risk of developing mental disorders, particularly post-traumatic stress disorder and depression [6, 7]. Living in areas with high rates of violence has been associated with poorer physical and psychological health [8, 9]. Individuals living in slums across Latin America frequently experience high levels of violence as a result of drug trafficking, organised crime, gang activity, and police interventions [10, 11]. These individuals are simultaneously exposed to other risk factors associated with poor mental health including poverty, limited education and employment opportunities, and reduced access to healthcare services [12]. 

Research on mental disorders in low- and middle-income countries (LMICs) is limited due to under-reporting and under-diagnosis, despite the fact that 80% of individuals with mental disorders reside in LMICs [13]. Evidence on mental health and the psychological health effects of violence are generally well-documented, with associations identified between violence and poor mental health outcomes [9, 12, 14, 15] However, systematic reviews on the effects of conflict and neighbourhood violence on mental health have identified a weak evidence base, methodological limitations, and that the causal pathways between violence and mental health are poorly understood, particularly in LMICs [9, 16–18] Meta-analyses by Fowler et al [14]. and McDonald & Richmond [15] found consistent links between community violence exposure and higher rates of mental disorders. However, studies often measure violence through questionnaires, relying on self-reported data, therefore making findings susceptible to recall bias [19, 20]. Additionally, while cumulative exposure to violence is linked to worse health outcomes, few studies examine its long-term effects [20–24].

Neighbourhood violence may influence mental health and healthcare use through several interconnected pathways. Exposure to violent events can act as an acute psychological stressor, contributing to anxiety, depressive symptoms, and trauma-related responses [6, 8, 9]. Repeated exposure may also generate chronic fear, hypervigilance, and anticipatory stress, which can erode wellbeing over time [8, 9]. In addition to these psychological effects, violence can shape behaviour by restricting movement through public spaces, disrupting daily routines, and reducing individuals’ willingness or ability to seek care [8, 11]. As a result, changes in mental health-related primary healthcare use may reflect both increased mental health needs and altered care-seeking or access in response to insecurity [8, 12].

An estimated one fifth of Rio de Janeiro’s population live in slums known as *favelas* and one third of the population reside in low-income neighbourhoods that make up 17% of the city’s geography [25]. Violence in these settings is endemic due to organised crime and police operations, contributing to a homicide rate of 16.9 per 100,000 in 2020 [26, 27]. While homicide rates vary substantially across Brazilian cities and regions, Rio de Janeiro remains one of the country’s most prominent urban settings affected by chronic and highly visible forms of armed violence [28]. Against this backdrop, this study investigates how acute neighbourhood violent events affect mental health outcomes among individuals living in low income neighbourhoods in Rio de Janeiro, Brazil. Specifically, we examine whether recent exposure to nearby violent incidents is associated with changes in mental health-related primary healthcare use. To address this, we combine individual electronic health records with socioeconomic data, alongside detailed neighbourhood-level violence exposure data. By focussing on acute, time-specific violent events, we reduce potential confounding from unobserved, time-invariant contextual characteristics. Using longitudinal data and fixed-effects models, we aim to strengthen causal inference on mental health consequences of neighbourhood violence beyond its most visible outcomes such as homicide and physical injury.

## Methods

### Study design & population

This study is a retrospective cohort analysis of 1,358,943 primary healthcare users with linked socioeconomic and primary healthcare (PHC) records in Rio de Janeiro, Brazil. A month-year individual-level panel was constructed for all individuals (aged 0-100 years) registered with PHC up to 31 December 2016. Individuals were excluded if they lacked valid clinic identifiers (CNES codes), had implausible demographic data, or attended clinics without geocoded violence information. For further details on the population and cohort construction, see Appendix 1.

### Violence data & exposure

Violence exposure measures were constructed by combining neighbourhood-level data on violent events from two main sources: reports of drug faction shootouts from *Disque-Denúncia *[29] (crime hotline) and police operation data from the *Grupo de Estudos dos Novos Ilegalismos* (Study of New Illegalities Group; GENI). Violence data were available from January 2009 onwards, allowing complete measurement of exposure from the start of PHC follow-up in January 2010 and enabling construction of lagged exposure variables without left-censoring. Co-authors VP and JG assembled and geocoded the violent event data. Events were geocoded using official spatial data and cross-referenced with favela boundary datasets. Individuals were classified as exposed to neighbourhood violence if a violent event occurred within 250 m of the PHC clinic they attended (straight-line distance). For the analysis, an individual’s clinic was assigned based on the first PHC clinic they attended during the study using CNES codes to link to individual-level panel data. For further details on exposure variable construction and data validation, see Appendix 2.

### Outcomes

The outcome of interest was primary healthcare consultations for any mental disorder identified through International Classification of Disease (ICD-10) and International Classification of Primary Care (ICPC) Codes. The mental disorders or associated mental health conditions included were: substance abuse/dependence disorders (ICD-10 F10-F19; ICPC P15, P17-P19); psychotic syndromes (ICD-10 F20-F25, F28, F29; ICPC P29, P71-P73, P98, P99); mood affective disorders (ICD-10 F30-F34, F38, F39; ICPC P03, P76); neurotic, stress-related and somatoform disorders (anxiety disorders) (ICD-10 F40-F45, F48; ICPC P01, P02, P74, P79, P82); personality and behaviour disorders (ICD-10 F50, F51, F60-F63, F91-F94; ICPC P80, P86); suicide/associated outcomes (ICD-10  X60-X84, R45.851; ICPC P77) (Appendix 3) [30–32]. All relevant ICD-10 and ICPC codes were included in the analysis to capture any mental health-related contact with the health system, allowing for comorbidity across diagnostic categories [33]. Mental disorders did not include neurological and neurodevelopmental disorders [34–36].

### Statistical analyses

Descriptive statistics were reported based on whether individuals were ever classified as exposed to a violent event – defined as the occurrence of a violent event within 250 m (straight-line distance) of the first PHC clinic they attended – at any point during the study period. The total number of PHC consultations related to mental health outcomes were reported, along with rates per 100,000 person-years. Cohort characteristics by type of violence and person-years of the study population were reported.

Descriptive analyses and models predicting violence exposure rely on pooled individual characteristics and time-invariant measures of exposure (ever exposed during the study period) and are intended to characterise socioeconomic patterns of violence exposure rather than to estimate causal effects. In contrast, the primary analyses examining the association between neighbourhood violence and mental health consultations exploit the longitudinal panel structure of the data using individual fixed-effects models, allowing comparisons within individuals over time.

A Poisson regression model was used to estimate the likelihood of individuals being exposed to neighbourhood violence (for either type and by drug-gang and police violence; binary) based on key socioeconomic factors, including sex, race/skin colour, education, age, income, welfare claimant status, and health insurance status. Following this, a panel data analysis was conducted to examine the association between neighbourhood violence exposure and PHC consultations for mental health conditions. Fixed-effects Poisson regression models were employed to account for time-invariant individual characteristics and better isolate the relationship between local neighbourhood violence and PHC consultations for mental health conditions. This modelling strategy focuses on within-individual changes over time, examining how temporal variation in local violence exposure is associated with changes in an individual’s healthcare utilisation. Unlike multilevel modelling approaches that emphasise between-area differences, the fixed-effects framework was chosen to control for all time-invariant individual characteristics (observed and unobserved) while addressing our primary question about short-term changes in exposure and service use [37]. Under the assumption that no unmeasured time-varying confounders jointly influence both exposure to neighbourhood violence and mental health-related PHC use, the resulting estimates can be interpreted as consistent with a causal effect of short-term changes in local violence exposure.

The primary exposure variable categorised the number of violent events occurring in a given month-year into six groups: 0, 1, 2, 3, 4, and 5 or more events. This categorisation was used to reflect increasing intensity of violence exposure and to assess potential dose–response relationships between violence and mental health outcomes.

To explore the temporal relationship between neighbourhood violence exposure and healthcare use, lagged exposure variables were created. Individual lags from one to six months (L1–L6) were tested in separate regressions to capture both immediate and delayed responses to acute violent events. This lag structure reflects evidence that psychological distress and trauma-related symptoms may emerge gradually following exposure, and that individuals may delay seeking care due to stigma, avoidance, or logistical barriers [8, 38]. The six-month window was selected to represent a plausible short- to medium-term period over which violence-related changes in mental health and healthcare utilisation may manifest. Interaction terms between all seven individual- and household-level socioeconomic variables and the violence exposure variable were introduced in separate regressions to assess potential disparities in the association between violence exposure and mental health outcomes.

To address potential bias from unobserved heterogeneity in individual and clinic characteristics, we employed fixed-effects Poisson regression models for the main analyses. Individual fixed effects controlled for all time-invariant characteristics. Additionally, month and year fixed effects were included to adjust for temporal trends and seasonality. Clustered robust standard errors were applied at the individual level to address within-person correlation. Control variables included the number of health teams and the total number of appointments (winsorised). While four clinic-level covariates were included in the inverse probability of treatment weighting (IPTW) model, only two were adjusted for in the regression due to collinearity and weaker theoretical links to the outcome. This reflects the distinct purposes of weighting (balancing exposure groups) versus outcome modelling (estimating associations).

Results were reported as incidence rate ratios (IRRs) with 95% confidence intervals (CIs). The incidence rate ratios (IRRs) represent the relative change in the rate of primary healthcare consultations for mental health conditions associated with different levels of exposure to violent events in the local neighbourhood, compared to the reference category (not exposed to violence in that month-year; zero). Clinic covariates were expressed as continuous variables, and their effects are interpreted as IRRs, representing the relative change in the outcome rate associated with a one-unit increase in each predictor. Poisson regression was employed to model count outcomes, appropriate for assessing the frequency of mental health-related events over time.

### Sensitivity analyses

Several robustness checks were conducted. First, random-effects Poisson models were estimated to compare with fixed-effects results. Second, alternative exposure categorisations were tested (including 0–10 + events).

Inverse probability of treatment weighting (IPTW) was used to assess robustness to measured confounding. Stabilised weights were generated using a multinomial logistic model predicting violence exposure category from baseline individual sociodemographic characteristics, clinic-level covariates, and calendar time. Weighted Poisson models were then estimated using pooled and random-effects specifications, as fixed-effects Poisson models cannot accommodate probability weights in Stata. Although these models do not control for unobserved time-invariant heterogeneity, comparison with the primary fixed-effects results provided a complementary robustness assessment. See Appendix 4 for more details.

The fixed-effects specification was prioritised in the main analysis to control for unmeasured, time-invariant individual heterogeneity likely correlated with both violence exposure and healthcare utilisation.

## Results

**Fig. 1:**
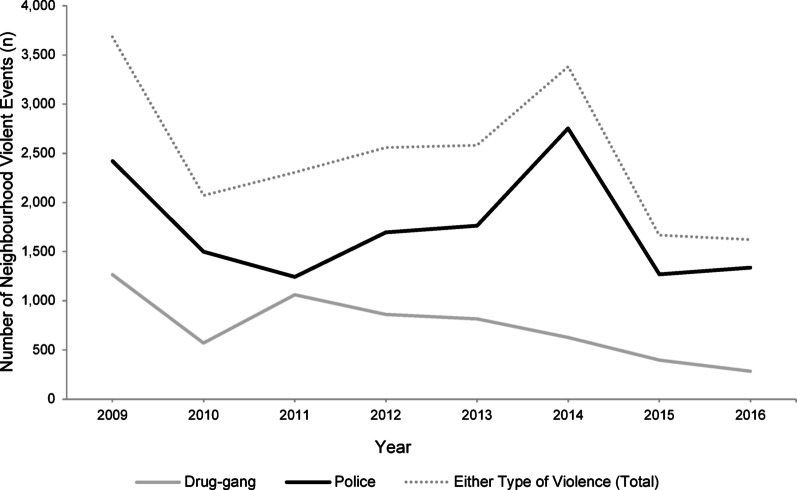
Annual trends of neighbourhood violent events by violence type, and drug-gang and police violence separately.

The cohort was composed of 1,358,943 individuals representing 3,712,094 person-years of observation (mean = 2.73 person-years per individual). Between January 2009 and December 2016, a total of 19,868 neighbourhood violent events were recorded. Of these events, 5,886 (29.6%) were drug-gang-related shootings and 13,982 (70.4%) were police-related shootings. The overall number of violent events peaked in 2009 (*n* = 3,683) and again in 2014 (*n* = 3,380), and gradually declined over time, reaching the lowest level in 2016 (*n* = 1,624) (Fig. 1). Police-related violence consistently accounted for the majority of events each year. Among the cohort, 67.2% were exposed to violence at least once during the study period (i.e., a violent event occurred within 250 m of their assigned primary healthcare clinic; Table 1). The distribution of demographic and socioeconomic characteristics was broadly similar between those exposed and unexposed, although differences were observed in race/skin colour and education (Table 1). Patterns were comparable when examining exposure to drug-gang and police violence separately.

**Fig. 2:**
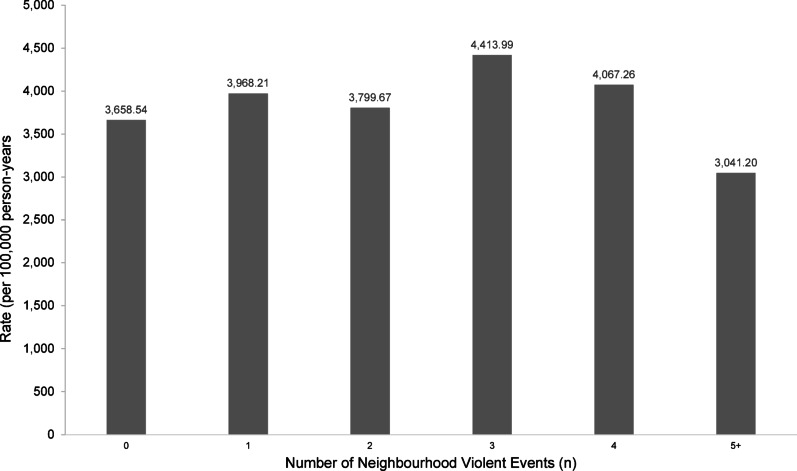
Mental health outcome rates by monthly violent event count (per 100,000 person-years) for all types of violence.

Crude consultation rates per 100,000 person-years were calculated by monthly violence exposure category (Fig. 2). The highest consultation rate was observed in months with three recorded violent events (4,413.99 per 100,000 person-years), compared with months with one event (3,968.21 per 100,000 person-years). Rates remained relatively stable across months with zero, two, and four events, but declined again at the highest exposure level – five or more events – where the consultation rate dropped to 3,041.20 per 100,000 person-years.

In adjusted cross-sectional Poisson regression models (Table 2), females had a 2% higher likelihood of exposure to either type of neighbourhood violence (IRR: 1.02; 95% CI: 1.01–1.02; *p* < 0.001), with a slightly higher likelihood of exposure to both drug-gang and police violence. Black individuals were 8% more likely to be exposed to violence compared to White individuals (IRR: 1.08; 95% CI: 1.07–1.09; *p* < 0.001), including a 12% higher exposure to drug-gang violence and 7% to police violence. Individuals with high school or higher education were 19% less likely to be exposed to violence overall (IRR: 0.81; 95% CI: 0.80–0.82; *p* < 0.001), with larger reductions for police violence (23%) than for drug-gang violence (3%).

Young adults (19–34 years) were 9% more likely to be exposed to any type of violence than youth (0–18 years) (IRR: 1.09; 95% CI: 1.08–1.10; *p* < 0.001), while older adults (65 + years) were 11% more likely to be exposed to drug-gang violence but 3% less to police violence. Compared to lower-income individuals, those with higher household income (earning two or more minimum salaries) were 22% less likely to be exposed to drug-gang violence (IRR: 0.78; 95% CI: 0.77–0.80; *p* < 0.001), but 9% more likely to be exposed to police violence (IRR: 1.09; 95% CI: 1.07–1.11; *p* < 0.001). Private health insurance was associated with consistently lower exposure across all types, particularly police violence (16% lower; IRR: 0.84; 95% CI: 0.83–0.85; *p* < 0.001).

**Table 1:** Cohort characteristics if ever exposed to violence in the study period (by either type of violence, and drug-gang and police violence separately).

Characteristics	Ever exposed								
	Either type of violence (*n*; %)			Drug-gang violence (*n*; %)			Police violence (*n*; %)		
	No	Yes	Total	No	Yes	Total	No	Yes	Total
**Sex**									
Male	175,499 (39.4%)	351,079 (38.4%)	526,578 (38.7%)	236,244 (39.2%)	290,334 (38.4%)	526,578 (38.7%)	207,531 (39.2%)	319,047 (38.5%)	526,578 (38.7%)
Female	270,319 (60.6%)	562,046 (61.6%)	832,365 (61.3%)	366,738 (60.8%)	465,627 (61.6%)	832,365 (61.3%)	321,998 (60.8%)	510,367 (61.5%)	832,365 (61.3%)
**Race/skin colour**									
White	151,910 (34.1%)	309,316 (33.9%)	461,226 (33.9%)	209,445 (34.7%)	251,781 (33.3%)	461,226 (33.9%)	179,995 (34.0%)	281,231 (33.9%)	461,226 (33.9%)
Black	52,536 (11.8%)	120,773 (13.2%)	173,309 (12.8%)	70,395 (11.7%)	102,914 (13.6%)	173,309 (12.8%)	63,908 (12.1%)	109,401 (13.2%)	173,309 (12.8%)
Pardo (mixed race)	236,368 (53.0%)	449,162 (49.2%)	685,530 (50.4%)	314,239 (52.1%)	371,291 (49.1%)	685,530 (50.4%)	279,661 (52.8%)	405,869 (48.9%)	685,530 (50.4%)
Amarelo (Asian), Indigenous, other or unknown (missing)	5,004 (1.1%)	33,874 (3.7%)	38,878 (2.9%)	8,903 (1.5%)	29,975 (4.0%)	38,878 (2.9%)	5,965 (1.1%)	32,913 (4.0%)	38,878 (2.9%)
**Education**									
None/pre-school/literacy class	79,457 (17.8%)	136,041 (14.9%)	215,498 (15.9%)	107,049 (17.8%)	108,449 (14.3%)	215,498 (15.9%)	93,170 (17.6%)	122,328 (14.7%)	215,498 (15.9%)
Elementary school	185,798 (41.7%)	349,304 (38.3%)	535,102 (39.4%)	240,702 (39.9%)	294,400 (38.9%)	535,102 (39.4%)	221,575 (41.8%)	313,527 (37.8%)	535,102 (39.4%)
High school or higher education	154,120 (34.6%)	255,545 (28.0%)	409,665 (30.1%)	199,699 (33.1%)	209,966 (27.8%)	409,665 (30.1%)	183,864 (34.7%)	225,801 (27.2%)	409,665 (30.1%)
None reported (missing)	26,443 (5.9%)	172,235 (18.9%)	198,678 (14.6%)	55,532 (9.2%)	143,146 (18.9%)	198,678 (14.6%)	30,920 (5.8%)	167,758 (20.2%)	198,678 (14.6%)
**Age group (years)**									
0–18	113,561 (25.5%)	218,071 (23.9%)	331,632 (24.4%)	153,809 (25.5%)	177,823 (23.5%)	331,632 (24.4%)	132,386 (25.0%)	199,246 (24.0%)	331,632 (24.4%)
19–34	90,552 (20.3%)	185,346 (20.3%)	275,898 (20.3%)	123,698 (20.5%)	152,200 (20.1%)	275,898 (20.3%)	106,696 (20.1%)	169,202 (20.4%)	275,898 (20.3%)
35–54	113,440 (25.4%)	234,037 (25.6%)	347,477 (25.6%)	152,805 (25.3%)	194,672 (25.8%)	347,477 (25.6%)	134,748 (25.4%)	212,729 (25.6%)	347,477 (25.6%)
55–64	59,371 (13.3%)	126,065 (13.8%)	185,436 (13.6%)	79,536 (13.2%)	105,900 (14.0%)	185,436 (13.6%)	71,905 (13.6%)	113,531 (13.7%)	185,436 (13.6%)
65+	68,894 (15.5%)	149,606 (16.4%)	218,500 (16.1%)	93,134 (15.4%)	125,366 (16.6%)	218,500 (16.1%)	83,794 (15.8%)	134,706 (16.2%)	218,500 (16.1%)
**Household income**									
Some salary reported to less than full minimum salary	56,056 (12.6%)	103,675 (11.4%)	159,731 (11.8%)	72,686 (12.1%)	87,045 (11.5%)	159,731 (11.8%)	66,402 (12.5%)	93,329 (11.3%)	159,731 (11.8%)
More than one minimum salary but less than two minimum salaries	106,080 (23.8%)	193,460 (21.2%)	299,540 (22.0%)	135,544 (22.5%)	163,996 (21.7%)	299,540 (22.0%)	128,132 (24.2%)	171,408 (20.7%)	299,540 (22.0%)
Two minimum salaries or more	31,892 (7.2%)	50,504 (5.5%)	82,396 (6.1%)	43,923 (7.3%)	38,473 (5.1%)	82,396 (6.1%)	36,889 (7.0%)	45,507 (5.5%)	82,396 (6.1%)
No salary reported (unknown or missing)	251,790 (56.5%)	565,486 (61.9%)	817,276 (60.1%)	350,829 (58.2%)	466,447 (61.7%)	817,276 (60.1%)	298,106 (56.3%)	519,170 (62.6%)	817,276 (60.1%)
**Registered with private health insurance**									
No	417,800 (93.7%)	863,851 (94.6%)	1,281,651 (94.3%)	567,090 (94.0%)	714,561 (94.5%)	1,281,651 (94.3%)	496,997 (93.9%)	784,654 (94.6%)	1,281,651 (94.3%)
Yes	28,018 (6.3%)	49,274 (5.4%)	77,292 (5.7%)	35,892 (6.0%)	41,400 (5.5%)	77,292 (5.7%)	32,532 (6.1%)	44,760 (5.4%)	77,292 (5.7%)
**Bolsa família-claiming family**									
No	385,932 (86.6%)	792,502 (86.8%)	1,178,434 (86.7%)	525,703 (87.2%)	652,731 (86.3%)	1,178,434 (86.7%)	460,119 (86.9%)	718,315 (86.6%)	1,178,434 (86.7%)
Yes	59,886 (13.4%)	120,623 (13.2%)	180,509 (13.3%)	77,279 (12.8%)	103,230 (13.7%)	180,509 (13.3%)	69,410 (13.1%)	111,099 (13.4%)	180,509 (13.3%)
***N***	***445***,***818 (32.8%)***	***913***,***125 (67.2%)***	***1***,***358***,***943 (100.0%)***	***602***,***982 (44.4%)***	***755***,***961 (55.6%)***	***1***,***358***,***943 (100.0%)***	***529***,***529 (39.0%)***	***829***,***414 (61.0%)***	***1***,***358***,***943 (100.0%)***

**Table 2:** Adjusted incidence rate ratios from poisson regression models of binary neighbourhood violence exposure by sociodemographic factors (by either type of violence, and drug-gang and police violence separately)

Characteristics	Either type of violence (*n*; %)			Drug-gang violence (*n*; %)			Police violence (*n*; %)		
	IRR		95% CI	IRR		95% CI	IRR		95% CI
**Sex**									
Male	1 (ref)	–	–	1 (ref)	–	–	1 (ref)	–	–
Female	1.02	***	(1.01–1.02)	1.03	***	(1.02–1.04)	1.02	***	(1.01–1.02)
**Race/skin colour**									
White	1 (ref)	–	–	1 (ref)	–	–	1 (ref)	–	–
Black	1.08	***	(1.07–1.09)	1.12	***	(1.11–1.13)	1.07	***	(1.06–1.08)
Pardo (mixed race)	1.00		(0.99–1.01)	1.03	***	(1.03–1.04)	0.99	*	(0.99–1.00)
Amarelo (Asian), Indigenous, other or unknown (missing)	1.05	***	(1.04–1.06)	1.07	***	(1.06–1.09)	1.05	***	(1.03–1.06)
**Education**									
None/pre-school/literacy class	1 (ref)	–	–	1 (ref)	–	–	1 (ref)	–	–
Elementary school	0.97	***	(0.96–0.97)	0.98	***	(0.97–0.99)	0.96	***	(0.95–0.97)
High school or higher education	0.81	***	(0.80–0.82)	0.97	***	(0.96–0.98)	0.77	***	(0.76–0.77)
None reported (missing)	1.45	***	(1.43–1.46)	1.15	***	(1.13–1.16)	1.50	***	(1.49–1.52)
**Age group (years)**									
0–18	1 (ref)	–	–	1 (ref)	–	–	1 (ref)	–	–
19–34	1.09	***	(1.08–1.10)	1.01		(1.00–1.02)	1.11	***	(1.10–1.12)
35–54	1.06	***	(1.05–1.07)	1.03	***	(1.02–1.04)	1.07	***	(1.06–1.08)
55–64	1.03	***	(1.03–1.04)	1.07	***	(1.06–1.08)	1.03	***	(1.02–1.04)
65+	1.00		(0.99–1.01)	1.11	***	(1.10–1.12)	0.97	***	(0.97–0.98)
**Household income**									
Some salary reported to less than full minimum salary	1 (ref)	–	–	1 (ref)	–	–	1 (ref)	–	–
More than one minimum salary but less than two minimum salaries	0.99	*	(0.98–1.00)	0.98	**	(0.97–0.99)	0.99		(0.98–1.00)
Two minimum salaries or more	1.02	**	(1.01–1.04)	0.78	***	(0.77–0.80)	1.09	***	(1.07–1.11)
No salary reported (unknown or missing)	1.02	***	(1.01–1.03)	1.02	**	(1.01–1.03)	1.04	***	(1.03–1.05)
**Registered with private health insurance**									
No	1 (ref)	–	–	1 (ref)	–	–	1 (ref)	–	–
Yes	0.86	***	(0.85–0.87)	0.94	***	(0.93–0.95)	0.84	***	(0.83–0.85)
**Bolsa família-claiming family**									
No	1 (ref)	–	–	1 (ref)	–	–	1 (ref)	–	–
Yes	0.99	***	(0.98–0.99)	0.91	***	(0.91–0.92)	1.00		(0.99–1.01)
***N***	*1*,*358*,*943*			*1*,*358*,*943*			*1*,*358*,*943*		

IRR – Incidence Rate Ratios; 95% CI – 95% Confidence Intervals. Separate Poisson regression models of cross-sectional cohort, adjusted for sex, race/skin colour, education level, age group, household income, private health insurance, Bolsa Família-receiving family. *p < 0.05; **p < 0.01; *** p < 0.001

### Panel regression analyses

In fixed-effects Poisson regression models, the number of neighbourhood violent events (per month) of any type was significantly associated with PHC consultations for mental health conditions (Table 3; full regression output in Appendix 5). Exposure to two and three neighbourhood violent events in a month was associated with a 5% (IRR: 1.05; 95% CI: 1.02–1.07; *p* < 0.001) and 8% (IRR: 1.08; 95% CI: 1.04–1.12; *p* < 0.001) increase in PHC consultations for mental health conditions, respectively. However, five or more violent events in a month was associated with a 7% decrease (IRR: 0.93; 95% CI: 0.89–0.97; *p* < 0.001) in consultations for mental health conditions.

When distinguishing by type of violence, different patterns emerged. Drug-gang-related violence was associated with a significant increase in consultations at exposure to three events per month (IRR: 1.13; 95% CI: 1.04–1.23; *p* < 0.01), but not at other violence levels. In contrast, police-related violence was linked to increased consultations at both two (IRR: 1.04; 95% CI: 1.01–1.06; *p* < 0.05) and three violent events per month (IRR: 1.07; 95% CI: 1.04–1.11; *p* < 0.001). At higher exposure levels, the direction of effect reversed; four drug-gang events in a month were associated with a 25% decrease in consultations (IRR: 0.75; 95% CI: 0.62–0.92; *p* < 0.01), and five or more police-related events were linked to a 9% decrease (IRR: 0.91; 95% CI: 0.87–0.96; *p* < 0.001).

**Fig. 3:**
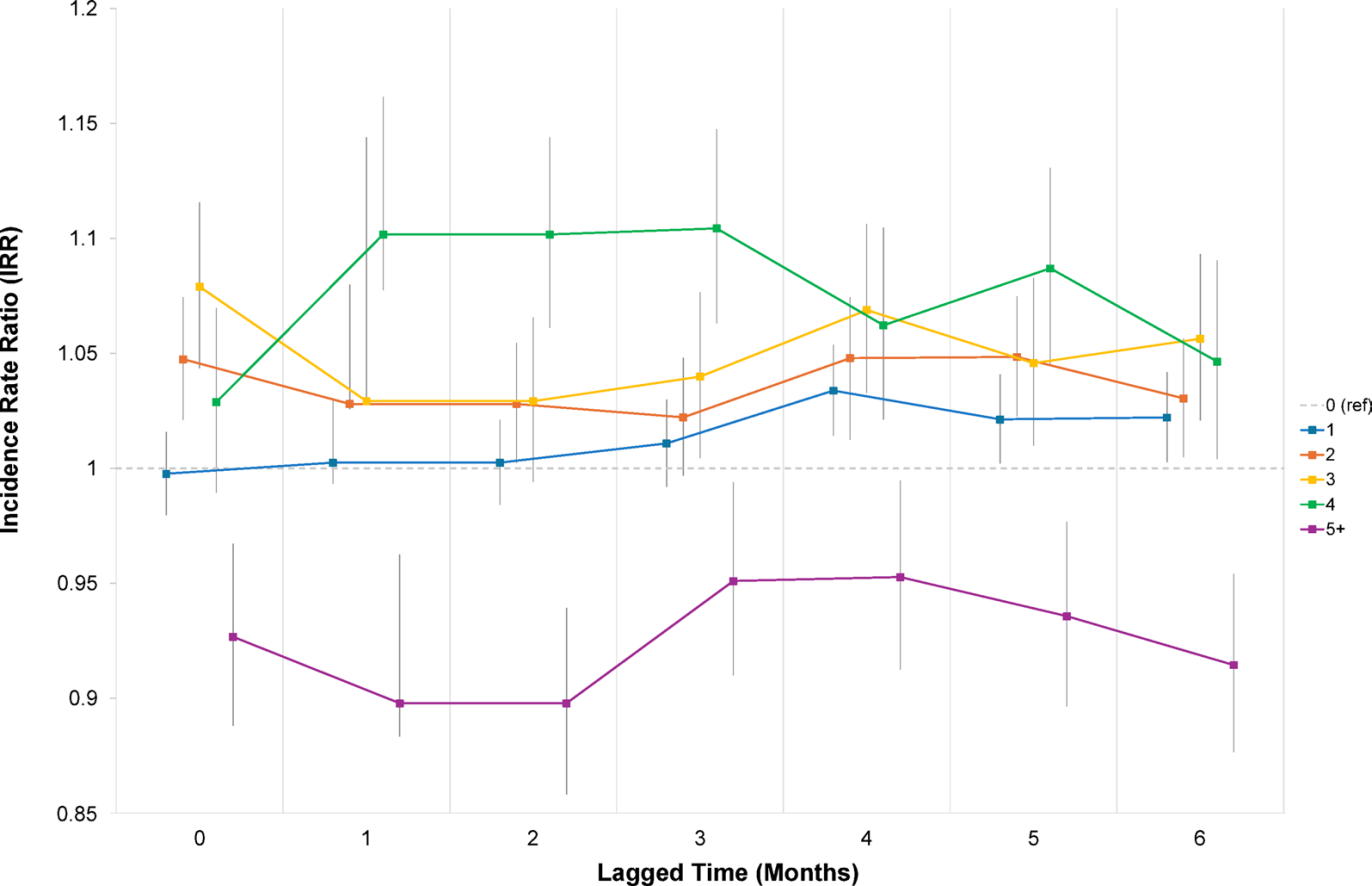
Incidence rate ratios from separate fixed effects poisson panel regression models examining one- to six-month lagged effects of neighbourhood violence (categorised as 0, 1, 2, 3, 4, and 5 + violent events) on primary healthcare consultations for mental health, including the primary non-lagged effects for reference. Adjusted for continuous clinic covariates (number of health teams and number of appointments), year, and month (see Appendices 5–10 for full regression outputs).

Examining lagged effects (Fig. 3; Appendices 6–11), distinct patterns emerged across levels of violence exposure. Moderate exposure to violence (two to four violent events per month) was consistently linked to an elevated risk of mental health consultations, particularly at 2–4 months after exposure. For example, at a two-month lag, exposure to two events was associated with a 3% increase in consultations (IRR: 1.03; 95% CI: 1.00–1.05; *p* < 0.05), and exposure to four events was linked to a 10% increase (IRR: 1.10; 95% CI: 1.06–1.14; *p* < 0.001). This trend persisted at three months (e.g., four events: IRR: 1.10; 95% CI: 1.06–1.15; *p* < 0.001) and four months (e.g., two events: IRR: 1.05; 95% CI: 1.02–1.07; *p* < 0.001; four events: IRR: 1.06; 95% CI: 1.02–1.10; *p* < 0.01), with similar significant associations. In contrast, very high exposure (five or more events per month) was generally associated with a decrease in mental health consultations across all lag periods, with the strongest effect observed at a two-month lag (IRR: 0.90; 95% CI: 0.86–0.94; *p* < 0.001).

Interactions between socioeconomic variables – including sex, race/skin colour, *Bolsa Família*-claiming family, private health insurance registration, and household income – and exposure to violence were generally not significant across any model specification, with the exception of age group and education level (Appendices 12–18). Individuals with high school or higher education exposed to the highest level of violence (5 + neighbourhood violent events in a month) had a 20% decrease in PHC consultations (IRR: 0.80; 95% CI: 0.68–0.94; *p* < 0.01) compared to individuals with no education/pre-school/literacy class in months with zero neighbourhood violent events (Appendix 14). Interaction effects between violence exposure and age group showed that individuals in all age groups experienced statistically significant increases in PHC consultations for mental health at the highest level of violence exposure compared to individuals aged 0–18 years in months with zero neighbourhood violent events (Appendix 15), indicating differential age responses rather than uniform increases across the cohort. Significant age-based differences were observed in the relationship between violence exposure and mental health consultations. At the highest exposure level (5 + violent events in a month), all adult age groups (19 + years) showed increased consultation rates, ranging from a 39% to 50% increase compared to unexposed individuals aged 0–18 years. The largest effect was observed among those aged 19–34 (IRR: 1.50; 95% CI: 1.16–1.95). At lower exposure levels, smaller but significant increases were also found, particularly among individuals aged 19–34 and 55–64.

### Sensitivity analyses

Sensitivity analyses supported the robustness of the primary findings. A random-effects model for any type of violence produced estimates consistent with the fixed-effects model, suggesting that unobserved individual heterogeneity did not substantially influence the results (Appendix 19). An alternative categorisation of violence exposure, including a more granular classification of 0–10 + violent events yielded no additional insights beyond the 0–5 + classification used in the main analysis (Appendix 20).

IPTW-weighted Poisson models, estimated using both pooled and random-effects specifications, produced effect estimates that were broadly consistent with the main fixed-effects regression (Appendices 21 and 22). Across violence exposure categories, incidence rate ratios remained directionally and substantively similar, particularly for moderate levels of exposure where the main associations were concentrated. Slightly stronger associations were observed in the weighted models at higher exposure levels, which may reflect additional control for clinic- and individual-level confounding not captured by fixed effects alone. These results suggest that the primary findings were not driven solely by observed baseline differences between exposure groups, and that unmeasured time-invariant individual heterogeneity (captured by fixed effects) remains a key source of confounding. Together, the findings from the weighted sensitivity analyses reinforce the validity and robustness of the main results.

**Table 3:** Incidence rate ratios from fixed effects poisson regression models examining the effect of neighbourhood violence on primary healthcare consultations for mental health for either type of violence, and drug-gang and police violence separately.

Characteristics	Either type of violence			Drug-gang violence			Police violence		
	IRR		95% CI	IRR		95% CI	IRR		95% CI
Number of neighbourhood violent events (per month)									
0	1 (ref)	–	–	1 (ref)	–	–	1 (ref)	–	–
1	1.00		(0.98–1.02)	1.00		(0.97–1.02)	0.99		(0.97–1.01)
2	1.05	***	(1.02–1.07)	1.01		(0.96–1.07)	1.04	*	(1.01–1.06)
3	1.08	***	(1.04–1.12)	1.13	**	(1.04–1.23)	1.07	***	(1.04–1.11)
4	1.03		(0.99–1.07)	0.75	**	(0.62–0.92)	1.00		(0.96–1.04)
5+	0.93	***	(0.89–0.97)	1.07		(0.90–1.26)	0.91	***	(0.87–0.96)
**Number of clinic health teams**	1.04	***	(1.03–1.06)	1.04	***	(1.03–1.06)	1.05	***	(1.03–1.06)
**Number of clinic appointments**	1.00	***	(1.00–1.00)	1.00	***	(1.00–1.00)	1.00	***	(1.00–1.00)
*Total observations (N)*	*1*,*384*,*805*			*1*,*384*,*805*			*1*,*384*,*805*		
*Total groups (N)*	*38*,*054*			*38*,*054*			*38*,*054*		

IRR – Incidence Rate Ratios; 95% CI – 95% Confidence Intervals. Separate fully adjusted fixed effects Poisson panel regression models per outcome (either type of violence, drug-gang violence, police violence);, adjusted for continuous clinic covariates (number of health teams and number of appointments), year, and month. *p < 0.05; **p < 0.01; *** p < 0.001

## Discussion

Neighbourhood violence exposure varied by demographic and socioeconomic factors. Females, Black individuals, and young adults faced a higher risk of exposure to violence, particularly to drug-gang violence. Higher education and private health insurance were linked to consistently lower violence exposure, especially to police violence. Exposure to two or three neighbourhood violent events in a month was associated with increased mental health consultations for both drug-gang and police-related violence. Five or more neighbourhood violent events per month were associated with a 7% decline in mental health consultations overall, with a larger reduction of 9% observed for police-related violence. Notably, increases in mental health consultations were observed not only immediately following exposure, but also at two- to four-month lags, indicating sustained effects over time. The relationship between violence exposure and mental health consultations was generally similar across socioeconomic groups; however, younger (19–34 years) and older (65 + years) adults had significantly higher increases in consultations, while individuals with higher education levels had significant decreases in consultations at high violence exposure levels.

The association between an increasing number of violent events and increased PHC consultations for mental health conditions aligns with existing global evidence on the detrimental impact of neighbourhood violence on population mental health [9, 12, 14, 18, 39–42]. Violence acts as a stressor, exacerbating psychological distress and increasing demand for mental health services. These findings suggest that beyond direct victimisation, broader neighbourhood violence in urban settings likely contributes to widespread mental health burdens, reinforcing the theory that chronic exposure to violence fosters sustained stress and trauma [9]. Individuals residing in high-violence areas may experience persistent anxiety and heightened psychological distress which can manifest as trauma- and stressor-related disorders such as post-traumatic stress symptoms, which are common in settings of repeated exposure to armed violence, necessitating greater mental health support [8]. However, the decline in consultations observed at the highest levels of violence exposure may reflect both demand- and supply-side barriers to accessing care. In contexts of extreme violence, fear of leaving home or travelling through unsafe areas may deter individuals from seeking healthcare, even when mental health needs remain high. At the same time, violence may disrupt service delivery by limiting clinic operations, reducing staff attendance, or prompting temporary closures. This pattern suggests that excessive neighbourhood violence not only harms mental health but also suppresses healthcare utilisation and availability, potentially exacerbating unmet needs in the most affected areas [8, 43–45].

The lagged effects of neighbourhood violence on mental health consultations suggest both immediate and delayed impacts. Exposure to two to four violent events per month was consistently associated with elevated consultation rates across several lag periods – particularly at two-, three-, and four-month lags – though not all estimates reached statistical significance. This pattern aligns with evidence that conditions such as post-traumatic stress disorder, anxiety, and depression often emerge gradually following trauma, and that individuals may delay seeking care until symptoms intensify. The absence of strong effects at a one-month lag could reflect delayed recognition of mental health needs, as well as stigma or logistical barriers to care-seeking in the short term [38, 46]. Interestingly, even exposure to one violent event per month showed small increases in consultations at longer lags, which may indicate that isolated incidents still trigger delayed psychological responses for some individuals.

Conversely, the decrease in consultations following exposure to five or more violent events may reflect an initial deterrent effect, where extreme violence heightens fear and reduces individuals’ willingness or ability to access care. However, the persistence of suppressed consultation rates may reflect more enduring disruptions, including longer-term interruptions to health service provision, system strain, community disengagement, and psychological desensitisation or demoralisation [8, 43–45]. In addition, competing risks such as injury, displacement, or death among highly exposed individuals may contribute to reduced primary care attendance. Finally, extreme violence may cluster within particular months rather than represent sustained exposure, raising the possibility that short-term spikes reflect temporal concentration rather than stable increases in violence intensity. These mechanisms are not mutually exclusive, and additional data would be required to disentangle them.

The findings indicate that exposure to neighbourhood violence is associated with increased primary healthcare consultations for mental health conditions, particularly among younger (19–34 years) and older (65 + years) adults. The former aligns with Brazilian literature showing that young adults in this age range are often both the main victims and perpetrators of violence, which may lead to worsening psychological health [47]. For older adults, the increased consultations may reflect heightened fear and vulnerability to stress-related mental health deterioration following exposure to neighbourhood violence and crime, as documented primarily in studies from high-income countries [48, 49]. Although no statistically significant interactions were observed for race/skin colour or sex, this should not be interpreted as evidence of the absence of social inequalities. Structural inequalities in Brazil shape both exposure to severe violence and patterns of healthcare utilisation. For example, Black and Pardo (mixed race) males are disproportionately affected by lethal violence and may be more likely to experience outcomes such as hospitalisation or death rather than primary care consultations [47, 50]. These patterns may reflect different pathways through which violence influences health outcomes and healthcare utilisation across social groups. The observed differences by education level may reflect how socioeconomic status influences patterns of healthcare utilisation in the context of violence. Individuals with higher education showed reduced primary healthcare consultations for mental health conditions at high levels of violence exposure, which could relate to differences in access to alternative support systems, mental health literacy, or perceived stigma in seeking care. While the mechanisms remain unclear, these patterns are consistent with evidence suggesting that more educated individuals may be better positioned to seek private or informal support or may underutilise primary care services for mental health needs [38, 46, 51]. Overall, these findings suggest that the mental health consequences of neighbourhood violence are socially patterned, with age and socioeconomic position influencing both vulnerability and healthcare-seeking responses.

This study has several strengths. First, the use of a large, population-level panel dataset comprising over 1.3 million individuals provides extensive coverage of PHC users. The longitudinal design, spanning from 2010 to 2016, allows for the examination of trends over time and enables the assessment of both short- and long-term associations between neighbourhood violence and mental health outcomes. By leveraging individual fixed effects regression, this study effectively controls for time-invariant individual-level confounders, reducing the risk of bias from unobserved heterogeneity and strengthening causal inference. Another key advantage of this study is the use of rich geocoded violence data, which enables precise linkage between neighbourhood violence exposure and health outcomes at the individual level. This spatial dimension provides a more accurate assessment of how local violence affects access to and use of PHC services, and consequently mental health outcomes. Additionally, the dataset includes clinic-level covariates allowing for the adjustment of facility-level factors that may influence healthcare access and quality. 

Several limitations should be considered when interpreting these findings. The outcome measure aggregates multiple diagnostic categories, including mood disorders, anxiety and stress-related disorders, psychotic syndromes, substance use disorders, and suicide-related outcomes. These conditions may respond differently to violence exposure, and combining them may obscure important heterogeneity in short- and longer-term effects. However, aggregation was chosen to capture overall mental health-related service use and to accommodate comorbidity and variability in routine diagnostic coding. In addition, the exposure metric captures discrete, geolocated violent events and does not fully reflect chronic and structural dimensions of violence described in sociological and public health research, such as persistent armed presence, restrictions on mobility, and ongoing insecurity in affected neighbourhoods [52–54]. These unmeasured forms of exposure may contribute to cumulative psychological stress and barriers to healthcare access that are not fully represented in our estimates. Violence may also affect health indirectly by restricting mobility, disrupting employment, and altering daily routines in affected communities [52–56]. Such constraints can limit individuals’ ability to seek care and contribute to chronic stress, but could not be measured using the available administrative and geospatial data. These pathways likely represent additional mechanisms through which violence influences health and healthcare utilisation that are not fully captured in the estimates.

A further limitation relates to exposure misclassification. Violence exposure was assigned based on events occurring within 250 m of the first registered PHC clinic attended, which may not fully reflect individuals’ residential environments or actual patterns of healthcare use. The assumption that individuals consistently attended the same clinic throughout follow-up may not capture real-world behaviour, as patients may switch facilities or avoid seeking care during periods of heightened insecurity. Such measurement error would likely attenuate estimated associations; however, clinic avoidance or changes in care-seeking behaviour during periods of extreme violence may also contribute to reduced observed consultation rates. We were also unable to obtain systematic data on temporary clinic closures or service disruptions related to violent events. These measurement challenges are compounded by the fact that armed confrontations in Rio de Janeiro can lead to short-term interruptions in primary healthcare service provision, including reduced clinic opening hours, staff absenteeism, or temporary closures. Consequently, observed changes in mental health consultations may reflect both shifts in psychological distress and fluctuations in healthcare availability. The estimated associations should therefore be interpreted as capturing the combined effects of violence on healthcare demand and constraints on access. Although clinic-level covariates were included to partially account for variations in service supply, residual confounding related to unmeasured service disruptions may remain. Furthermore, the true effects of violence on PHC use are likely underestimated, as violence is highly prevalent in Rio de Janeiro and the exposure measure captures only neighbourhood-level shootings, most of which were police-related. This excludes other important forms of violence, particularly domestic and sexual violence occurring within households, which follow different patterns, have distinct policy and service responses, and warrant separate investigation [57, 58].

Another consideration concerns the lack of time-varying socioeconomic measures. Changes in individuals’ socioeconomic status over time – such as shifts in income, employment, or education – could not be incorporated into the fixed-effects models and may therefore introduce bias if associated with both violence exposure and mental health service use. Sensitivity analyses using random-effects models that included baseline socioeconomic covariates produced similar results, supporting the robustness of the main findings. Further, mental health consultations represent healthcare utilisation and may not fully capture underlying mental health status and clinical incidence. In routine primary care data, formal psychiatric diagnoses may be inconsistently coded or under-recorded. Mental health-related consultations therefore provide a pragmatic and policy-relevant indicator of psychological distress and service demand, particularly in low-resource settings. The findings should be interpreted as reflecting changes in mental health-related service use, which may arise from both variation in need and constraints on access. Given that the study population consisted of low-income individuals registered with primary healthcare services, the findings may not be fully generalisable to higher-income populations, individuals who primarily use private healthcare, or residents of urban settings with different patterns of violence exposure and healthcare organisation. However, the results provide important insights for cities characterised by high levels of socioeconomic inequality, spatially concentrated violence, and reliance on primary care as the main entry point to mental health services.

This analysis was designed to estimate how short-term changes in exposure to nearby violent events are associated with changes in mental health-related primary healthcare use within the same individuals over time. It does not aim to estimate broader contextual effects of neighbourhood environments or the institutional dynamics of health service provision. As such, the findings should be interpreted as reflecting within-individual responses to temporal fluctuations in local violence rather than comprehensive neighbourhood-level or system-level impacts.

The findings of this study underscore the need to strengthen mental health responses in urban settings experiencing persistent violence. Mental health services should be expanded and better integrated within primary healthcare systems, particularly in neighbourhoods where exposure to violence is frequent. Allocating resources based on local patterns of violence exposure may help ensure that healthcare systems are adequately prepared to respond to fluctuations in demand for mental health consultations. Ensuring continuity of care in high-risk areas is especially important, as insecurity may disrupt mobility and access to services. Strengthening community-based psychosocial support and outreach may also help mitigate delayed care-seeking and unmet mental health needs in violence-affected populations.

Beyond health system responses, addressing the structural drivers of violence is essential for improving long-term population mental health. Comprehensive violence prevention strategies targeting socioeconomic inequality, unemployment, and social exclusion are critical. Community-based programmes such as Cure Violence Chicago [59] and Safe Streets Baltimore [60] in the United States provide instructive examples of outreach and conflict mediation initiatives that have contributed to reductions in shootings and homicides in disadvantaged urban areas. At the global level, the United Nation’s Youth, Peace and Security Programming Handbook [61] further emphasises the importance of community-based, youth-led approaches that integrate psychosocial support and address the underlying drivers of violence. Similarly, the Pelotas Pact for Peace, a city-led initiative in southern Brazil, exemplifies an integrated public health and criminal justice strategy to reduce violence in urban settings, achieving a 9% reduction in homicides and a 7% decrease in robberies while also investing in health and education to address underlying drivers of violence [62].

Additionally, there must be a concerted effort to reform policing strategies such as the *Unidade de Polícia Pacificadora* (Pacifying Police Unit) to reduce the incidence of violence stemming from police operations, ensuring that law enforcement is part of the solution, rather than exacerbating the problem [63, 64]. For example, the Group Violence Intervention strategy in the United States, previously successful as part of the Operation Ceasefire in the 1990s, focussed on community and problem-oriented, rather than incident-oriented policing, led to homicide reductions between 30% and 60%. Experiences from countries including Colombia and South Africa further highlight the importance of multisectoral initiatives that integrate data-driven policing, community outreach, and social policy interventions to address concentrated urban violence [65]. Overall, coordinated action across health, social, and public security sectors is essential to create safer urban environments and mitigate the long-term mental health consequences of chronic violence exposure.

## Conclusion

Neighbourhood violence in Rio de Janeiro was associated with increased use of PHC for mental health conditions. Moderate exposure (two to three violent events per month) was linked to higher consultation rates, while at the highest levels of violence (five or more events), healthcare use was suppressed, possibly due to access barriers or service disruptions. Increased mental health consultations were also observed two to four months after exposure, suggesting lasting impacts on psychological well-being. These findings underscore the mental health burden of chronic violence in urban settings, and highlight the need for timely, accessible, and sustained mental health support in communities affected by ongoing violence.

## Data Availability

The datasets generated and analysed during the current study are not publicly available due to the confidentiality of the linked data. They are available from the corresponding author on reasonable request and following approval from CONEP and CEP.

## Appendix

### Appendix 1. Cohort construction and data cleaning steps

PHC registration records of 3,732,630 individuals from the *Ficha de Cadastro Domiciliar e Territorial* (Household and Territorial Registration Form; FichaA) from the Brazilian Ministry of Health [66], were obtained and linked to PHC electronic medical records from the Primary Care Assessment Tool (PCAT) dataset covering the period 1 January 2010 to 31 December 2016. The datasets were linked via a combination of deterministic and probabilistic approaches, with details published elsewhere [67]. Individuals registered up to and including 31 December 2016 were included in the cohort. PHC services in Rio de Janeiro primarily serve residents of low-income neighbourhoods, therefore, the study population predominantly consisted of individuals of lower income residing in poorer neighbourhoods and/or favelas in the city, where violence levels are typically higher.

From PHC registration and utilisation records (FichaA and PCAT), individuals’ socioeconomic characteristics were collected and used as covariates in sensitivity analyses. These individual- and household-level covariates were included to control for any potential confounders that could be associated with neighbourhood violence exposure and mental health outcomes. Socioeconomic status is strongly associated with neighbourhood violence exposure and mental health outcomes. Individuals of lower-income, lower educational attainment and of non-White race and/or ethnicity often reside in the least socially cohesive neighbourhoods with high levels of poverty and crime, increasing the risk of experiencing or being affected by violence [68–71]. Similarly, socially disadvantaged populations are disproportionately affected by poor mental health [72].

Individual-level covariates included sex (male, female); race/skin colour (based on self-reported skin colour in Brazil – Black, White, Pardo [mixed race], Amarelo [Asian], Indigenous, other or unknown [missing]); highest educational attainment (none/pre-school/literacy class, elementary school, high school or higher education, none reported [missing]); and age group in years (0–18, 19–34, 35–54, 55–64, 65+). Household-level covariates included household income (some salary reported to less than full minimum salary, more than one minimum salary but less than two minimum salaries, two minimum salaries or more, no salary reported [unknown or missing]); registered with private healthcare insurance (yes, no); and whether the family receives Bolsa Família—a conditional cash transfer programme for families earning below US$70 per day (yes, no).

A month-year individual-level panel was constructed for all individuals (aged 0-100 years) registered with PHC up to 31 December 2016. All individuals that registered with PHC between 1 January 2010 to 31 December 2016 were included in the cohort. There were 3,732,630 individuals in the original dataset of registered individuals, of which 2,315,677 were removed; 509 died before the start of the observation period (1 January 2010), 583,197 individuals registered after the end of the observation period (31 December 2016), 78 were removed due to missing data on sex, 457 and 6 individuals removed as their dates of birth were before 1 January 1916 and after 31 December 2016, respectively, and 1,775,020 individuals were dropped due to missing data on *Cadastro Nacional de Estabelecimentos de Saúde* (National Registry of Health Establishments; CNES) which identify the PHC clinics individuals are registered to and access, necessary to identify exposure. A further 14,420 individuals were omitted from the dataset as the clinic they attended had missing violence data, resulting in a study population of 1,358,943 individuals. Individuals missing *Cadastro Nacional de Estabelecimentos de Saúde* (National Registry of Health Establishments; CNES) codes were excluded from the analysis, as the absence of clinic data meant their exposure to local violence could not be reliably assessed.

### Appendix 2. Construction and geocoding of neighbourhood violence data

#### Violence data

The cohort data was supplemented with information on local events of violence. Violence indicators were constructed by cross-referencing non-official crime statistics at the neighbourhood-level. Co-authors VP & JG constructed a dataset of violent events combining information from various sources using data from the *Disque-Denúncia *[29] (crime hotline) and a database containing information on police operations created by the *Grupo de Estudos dos Novos Ilegalismos* (Study of New Illegalities Group; GENI) where they scraped news and media publications from prominent police- and crime-related news publications including *O Dia*, *Extra*, and *Meia Hora* [73]. The *Disque-Denúncia* is a non-profit organisation that created a crime hotline and online platform where the public can anonymously report criminal activity, security issues, and public disorder, among other illicit activities. From *Disque-Denúncia*, all reports classified as “tiroteio entre facções” (shootouts between factions) were collected, including the date, location, and description of the event by colleagues at IEPS. To supplement the *Disque-Denúncia*, which focusses primarily on drug faction shootings, data on police operations collected by GENI at the *Universidade Federal Fluminense* (Federal Fluminense University; UFF) was requested online to broaden the scope of violence [74].

*Disque-Denúncia* attempts to mitigate any under- and overreporting by verifying anonymous reports and forwarding the relevant information to the appropriate authorities. The GENI/UFF data on police operations was created using multiple sources including new articles and social media platforms, and was cross-referenced with official government statistics to minimise errors and discrepancies, revealing a high level of consistency [75]. GENI/UFF acknowledge the presence of missing data, stating that they took steps to address this by using statistical methods and data imputation to estimate missing values, while also being transparent about the limitations the incomplete data may pose. The GENI/UFF data has been used in conjunction with violence data and reports from other organisations including the NGO, *Redes da Maré* and data lab, *Fogo Cruzado *[64]. Further, the GENI/UFF database has been used to inform Brazilian public policy and court cases, notably the ADFP n.635 addressing police raids in favelas [64, 76, 77].

To geocode violent events, colleagues at IEPS used an online map resource provided by the *Ministério Público do Estado do Rio de Janeiro* (Public Prosecutor’s Office of the State of Rio de Janeiro; MPRJ) [78] that covers the low-income neighbourhoods of the city of Rio de Janeiro. A dictionary of low-income neighbourhood names was created and compared with the address data from *Disque-Denúncia* and the GENI/UFF databases. If a neighbourhood could not be directly identified, the reported address was checked to determine if it was within a low-income neighbourhood. For these cases, checks were conducted using the same sources used by MPRJ, *Sistema de Assentamentos de Baixa Renda* (Low Income Settlement System; SABREN),^79^ information of favela boundaries and housing projects obtained from the Municipality of Rio de Janeiro,^25^ Wikimapia data [80], and Google Maps [81] to identify reported locations. This process enabled the standardisation of favela and low-income neighbourhood names in the violence databases, allowing them to be merged with the MPRJ spatial data.

Episodes of violence were linked to low-income neighbourhoods by matching the neighbourhood information from violence data sources to the dictionary of low-income neighbourhood names. Violent events that occurred outside the low-income neighbourhoods and/or did not have address or neighbourhood information for geocoding were disregarded. A total of 91% and 87% of shootouts between factions and police operations involving shootings, respectively, were geocoded and will be used in the analysis. Duplicate observations for the same neighbourhood-day violent events were removed.

Data on healthcare clinics including address data was obtained from CNES from the Rio de Janeiro Municipal Health Secretariat or publicly available data from the Rio de Janeiro City Hall for healthcare clinics that opened more recently [82]. A monthly clinic-level panel was constructed containing 204 PHC units that had at least one active PHC team between 2009 and 2016. The study dataset to be used in the analysis is composed of the individual-level month-year panel, in which clinic-month observations of violent events were merged onto through facility-level CNES codes.

#### Exposure

Individuals were classified as exposed to violence if they were registered at a PHC clinic located within 250 m of a neighbourhood where at least one violent event occurred. Violence exposure is herein referred to as neighbourhood violence.

To determine whether a clinic was exposed to violence, colleagues at IEPS utilised the proximity of clinics to violent events in low-income neighbourhoods. Clinics were considered exposed if they were located within a 250-metre radius (straight-line distance) of a low-income neighbourhood where geocoded violent events occurred. Monthly violence exposure for each clinic was calculated by merging the violent event dataset on to the clinic-level dataset. Violent events, including shootings, were linked to low-income neighbourhoods using geocoded data specifying addresses or neighbourhoods. Monthly counts of violent events in low-income neighbourhoods were aggregated for each clinic exposed, considering only those within the 250-metre buffer. Binary and count variables of violence exposure were generated based on whether a clinic was located within a violent event buffer zone (binary exposure per month-year) and how many times this occurs (count exposure per month-year). The clinic-level violence exposure dataset was then linked to the individual-level dataset using CNES codes to identify whether an individual was registered to and attended a PHC clinic that was located in a neighbourhood exposed to violence.

The neighbourhood violence data spans the years 2009 to 2016, while the individual panel data covers the period from 2010 to 2016. To ensure a comprehensive understanding of violence exposure, we include the 2009 neighbourhood violence data in our analysis. This approach allows us to account for the potential lagged effects of neighbourhood violence on individuals’ mental health outcomes. For individuals who attended more than one clinic during the study period, neighbourhood violence exposure was assigned based on the location of the first clinic they visited. This approach ensured consistent geographic exposure assignment across time, reduced misclassification from clinic-switching behavior, and helped mitigate bias from potentially endogenous clinic changes. Anchoring exposure to the first clinic allows us to capture the initial environment of care and its associated neighborhood violence context.

### Appendix 3. Mental disorder disaggregation by international classification of diseases (ICD-10) & international classification of primary care (ICPC) codes

**Table:**

Mental disorder classification	ICD-10 codes	ICPC codes & definitions
**Substance Abuse/Dependence Disorders** Mental and behavioural disorders due to use of alcohol Mental and behavioural disorders due to use of opioids Mental and behavioural disorders due to use of cannabinoids Mental and behavioural disorders due to use of sedatives or hypnotics Mental and behavioural disorders due to use of cocaine Mental and behavioural disorders due to use of other stimulants, including caffeine Mental and behavioural disorders due to use of hallucinogens Mental and behavioural disorders due to use of tobacco Mental and behavioural disorders due to use of volatile solvents Mental and behavioural disorders due to multiple drug use and use of other psychoactive substances	F10-F19	P15 Chronic alcohol abuse P17 Tobacco abuse P18 Medication abuse P19 Drug abuse
**Psychotic Syndromes** Schizophrenia Schizotypal disorder Persistent delusional disorders Acute and transient psychotic disorders Induced delusional disorder Schizoaffective disorders Other nonorganic psychotic disorders Unspecified nonorganic psychosis	F20-F25, F28, F29	P29 Psychological symptom/complt other P71 Organic psychosis disorder P72 Schizophrenia P73 Affective psychosis P98 Psychosis NOS/other P99 Psychological disorders, other
**Mood Affective Disorders** Manic episode Bipolar affective disorder Depressive episode Recurrent depressive disorder Persistent mood affective disorder Other mood affective disorders Unspecified mood affective disorder	F30-F34, F38, F39	P03 Feeling depressed P76 Depressive disorder
**Neurotic**,** Stress-related & Somatoform Disorders (Anxiety Disorders)** Phobic anxiety disorders Other anxiety disorders Obsessive-compulsive disorder Reaction to severe stress, and adjustment disorders Dissociative conversion disorders Somatoform disorders Other neurotic disorders	F40-F45, F48	P01 Feeling anxious/nervous/tense P02 Acute stress reaction P74 Anxiety disorder/anxiety state P79 Phobia/compulsive disorder P82 Post-traumatic stress disorder
**Personality & Behaviour Disorders** Specific personality disorders Mixed and other personality disorders Enduring personality changes, not attributable to brain damage and disease Habit and impulse disorders Conduct disorders Mixed disorders of conduct and emotions Emotional disorders with onset specific to childhood Disorders of social functioning with onset specific to childhood and adolescence Eating disorders Nonorganic sleep disorders	F50, F51, F60-F63, F91-F94	P80 Personality disorder P86 Anorexia nervosa/bulimia
**Suicide/Associated Outcomes** Intentional self-poisoning Intentional self-harm Suicidal ideation	X60-X69, X70-X84, R45.8	P77 Suicide/suicide attempt

### Appendix 4. Inverse probability of treatment weighting (IPTW) specification

To assess the robustness of the primary findings, additional model specifications were tested using IPTW. Stabilised IPTW weights were generated at the individual level using a multinomial logistic regression model, in which the categorical violence exposure variable was predicted by a range of baseline individual-level sociodemographic covariates (sex, race/skin colour, educational attainment, age group, household income, health insurance status, Bolsa Família status), clinic-level covariates (number of primary care teams, number of appointments, total clinic operating time, number of physicians), and calendar month and year. This approach aimed to balance observed characteristics across exposure groups and minimise bias due to non-random allocation of violence exposure.

Weighted Poisson regression models were estimated using the stabilised IPTW to examine whether adjusting for measured confounding at baseline influenced the association between violence exposure and primary healthcare consultations for mental health conditions. As fixed-effects Poisson models are incompatible with probability weights in Stata, these weighted models were conducted using pooled and random-effects Poisson regression specifications. While these approaches do not control for unobserved individual heterogeneity, they allow for explicit adjustment of time-invariant measured covariates and can accommodate IPTW. Comparing the results from these weighted models with the primary fixed-effects analysis provided a complementary robustness check.

### Appendix 5. Incidence rate ratios from fixed effects poisson regression models examining the effect of neighbourhood violence on primary healthcare consultations for mental health for either type of violence, and drug-gang and police violence separately

**Table:**

Characteristics	Either type of violence			Drug-gang violence				Police violence					
	IRR		95% CI	IRR			95% CI	IRR				95% CI	
**Number of neighbourhood violent events (per month)**													
0	1 (ref)	–	–		1 (ref)	–	–		1 (ref)	–			–
1	1.00		(0.98–1.02)		1.00		(0.97–1.02)		0.99				(0.97–1.01)
2	1.05	***	(1.02–1.07)		1.01		(0.96–1.07)		1.04	*			(1.01–1.06)
3	1.08	***	(1.04–1.12)		1.13	**	(1.04–1.23)		1.07	***			(1.04–1.11)
4	1.03		(0.99–1.07)		0.75	**	(0.62–0.92)		1.00				(0.96–1.04)
5+	0.93	***	(0.89–0.97)		1.07		(0.90–1.26)		0.91	***			(0.87–0.96)
**Number of clinic health teams**	1.04	***	(1.03–1.06)		1.04	***	(1.03–1.06)		1.05	***			(1.03–1.06)
**Number of clinic appointments**	1.00	***	(1.00–1.00)		1.00	***	(1.00–1.00)		1.00	***			(1.00–1.00)
**Month**													
1	1 (ref)	–	–	1 (ref)		–	–	1 (ref)			–	–	
2	0.97	*	(0.94–1.00)	0.97		*	(0.94–1.00)	0.97			*	(0.94–1.00)	
3	1.09	***	(1.06–1.12)	1.09		***	(1.06–1.12)	1.09			***	(1.06–1.12)	
4	1.03	*	(1.00–1.06)	1.02			(1.00–1.05)	1.03			*	(1.00–1.06)	
5	1.14	***	(1.11–1.18)	1.14		***	(1.11–1.18)	1.14			***	(1.11–1.18)	
6	1.21	***	(1.18–1.24)	1.21		***	(1.17–1.24)	1.21			***	(1.18–1.24)	
7	1.27	***	(1.24–1.31)	1.27		***	(1.23–1.30)	1.27			***	(1.24–1.30)	
8	1.35	***	(1.31–1.39)	1.35		***	(1.31–1.39)	1.35			***	(1.32–1.39)	
9	1.45	***	(1.41–1.49)	1.44		***	(1.41–1.48)	1.45			***	(1.41–1.48)	
10	1.40	***	(1.36–1.43)	1.39		***	(1.36–1.43)	1.40			***	(1.36–1.43)	
11	1.43	***	(1.39–1.46)	1.41		***	(1.38–1.45)	1.42			***	(1.38–1.46)	
12	1.45	***	(1.41–1.49)	1.45		***	(1.41–1.49)	1.45			***	(1.41–1.49)	
**Year**													
2010	1 (ref)	–	–	1 (ref)		–	–	1 (ref)			–	–	
2011	4.82		(0.64–36.07)	4.83			(0.65–36.13)	4.82				(0.64–36.03)	
2012	10.29	*	(1.38–76.87)	10.32		*	(1.38–77.12)	10.26			*	(1.37–76.66)	
2013	14.46	**	(1.94–108.08)	14.49		**	(1.94–108.29)	14.47			**	(1.94–108.11)	
2014	20.31	**	(2.72–151.80)	20.27		**	(2.71–151.51)	20.33			**	(2.72–151.91)	
2015	29.79	**	(3.99–222.62)	29.90		**	(4.00–223.40)	29.81			**	(3.99–222.72)	
2016	48.89	***	(6.54–365.28)	49.00		***	(6.56–366.11)	48.78			***	(6.53–364.50)	
*Total Observations (N)*	*1*,*384*,*805*				*1*,*384*,*805*				*1*,*384*,*805*				
*Total Groups (N)*	*38*,*054*				*38*,*054*				*38*,*054*				

IRR – Incidence Rate Ratios; 95% CI – 95% Confidence Intervals. Separate fully adjusted fixed effects Poisson panel regression models per outcome (either type of violence, drug-gang violence, police violence);, adjusted for continuous clinic covariates (number of health teams and number of appointments), year, and month. *p < 0.05; **p < 0.01; *** p < 0.001

### Appendix 6. Incidence rate ratios from a fixed effects poisson regression model examining the one-month lagged effects of neighbourhood violence on primary healthcare consultations for mental health

**Table:**

Characteristics	IRR		95% CI	
**Number of neighbourhood violent events (per month)**				
0	1 (ref)	–	–	
1	1.00		(0.98–1.01)	
2	1.05	***	(1.03–1.08)	
3	1.08	***	(1.04–1.12)	
4	1.05	*	(1.01–1.10)	
5+	0.95	*	(0.91–0.99)	
**One-month lagged number of neighbourhood violent events (per month)**				
0	1 (ref)	–	–	
1	0.99		(0.97–1.00)	
2	1.03	*	(1.00–1.05)	
3	1.00		(0.97–1.04)	
4	1.04		(1.00–1.08)	
5+	0.93	***	(0.89–0.97)	
**Number of clinic health teams**	1.05	***	(1.03–1.06)	
**Number of clinic appointments**	1.00	***	(1.00–1.00)	
**Month**				
January	1 (ref)	–	–	
February	0.97	*	(0.94–1.00)	
March	1.10	***	(1.07–1.13)	
April	1.04	**	(1.01–1.07)	
May	1.15	***	(1.12–1.19)	
June	1.22	***	(1.18–1.25)	
July	1.28	***	(1.24–1.31)	
August	1.36	***	(1.32–1.39)	
September	1.45	***	(1.41–1.49)	
October	1.40	***	(1.36–1.43)	
November	1.44	***	(1.40–1.47)	
December	1.45	***	(1.41–1.49)	
**Year**				
2010	1 (ref)	–	–	
2011	4.83		(0.65–36.20)	
2012	9.74	*	(1.30–72.86)	
2013	14.10	*	(1.89–105.45)	
2014	19.57	**	(2.62–146.36)	
2015	28.67	**	(3.83–214.37)	
2016	47.39	***	(6.34–354.39)	
*Total Observations (N)*	*1*,*328*,*580*			
*Total Groups (N)*	*36*,*147*			

IRR – Incidence Rate Ratios; 95% CI – 95% Confidence Intervals. Fixed effects Poisson regression, adjusted for continuous clinic covariates (number of health teams and number of appointments), year, and month. *p < 0.05; **p < 0.01; *** p < 0.001

### Appendix 7. Incidence rate ratios from a fixed effects poisson regression model examining the two-month lagged effects of neighbourhood violence on primary healthcare consultations for mental health

**Table:**

Characteristics	IRR		95% CI	
**Number of neighbourhood violent events (per month)**				
0	1 (ref)	–	–	
1	1.00		(0.98–1.02)	
2	1.05	***	(1.02–1.08)	
3	1.08	***	(1.04–1.12)	
4	1.06	**	(1.01–1.10)	
5+	0.94	**	(0.90–0.98)	
**Two-month lagged number of neighbourhood violent events (per month)**				
0	1 (ref)	–	–	
1	1.00		(0.98–1.02)	
2	1.03	*	(1.00–1.05)	
3	1.03		(0.99–1.07)	
4	1.10	***	(1.06–1.14)	
5+	0.90	***	(0.86–0.94)	
**Number of clinic health teams**	1.05	***	(1.04–1.06)	
**Number of clinic appointments**	1.00	***	(1.00–1.00)	
**Month**				
January	1 (ref)	–	–	
February	0.96	*	(0.93–0.99)	
March	1.10	***	(1.07–1.13)	
April	1.04	**	(1.01–1.07)	
May	1.16	***	(1.12–1.19)	
June	1.22	***	(1.18–1.25)	
July	1.27	***	(1.23–1.30)	
August	1.35	***	(1.31–1.38)	
September	1.44	***	(1.40–1.48)	
October	1.38	***	(1.35–1.42)	
November	1.42	***	(1.39–1.46)	
December	1.44	***	(1.40–1.48)	
**Year**				
2010	1 (ref)	–	–	
2011	4.63		(0.62–34.74)	
2012	9.24	*	(1.23–69.16)	
2013	13.66	*	(1.82–102.27)	
2014	18.65	**	(2.49–139.65)	
2015	27.37	**	(3.66–204.92)	
2016	45.39	***	(6.06–339.84)	
*Total Observations (N)*	*1*,*275*,*796*			
*Total Groups (N)*	*34*,*601*			

IRR – Incidence Rate Ratios; 95% CI – 95% Confidence Intervals. Fixed effects Poisson regression, adjusted for continuous clinic covariates (number of health teams and number of appointments), year, and month. *p < 0.05; **p < 0.01; *** p < 0.001

### Appendix 8. Incidence rate ratios from a fixed effects poisson regression model examining the three-month lagged effects of neighbourhood violence on primary healthcare consultations for mental health

**Table:**

Characteristics	IRR		95% CI	
**Number of neighbourhood violent events (per month)**				
0	1 (ref)	–	–	
1	1.00		(0.98–1.02)	
2	1.05	***	(1.02–1.08)	
3	1.08	***	(1.04–1.12)	
4	1.05	*	(1.01–1.09)	
5+	0.94	**	(0.90–0.98)	
**Three-month lagged number of neighbourhood violent events (per month)**				
0	1 (ref)	–	–	
1	1.01		(0.99–1.03)	
2	1.02		(1.00–1.05)	
3	1.04	*	(1.00–1.08)	
4	1.10	***	(1.06–1.15)	
5+	0.95	*	(0.91–0.99)	
**Number of clinic health teams**	1.05	***	(1.03–1.06)	
**Number of clinic appointments**	1.00	***	(1.00–1.00)	
**Month**				
January	1 (ref)	–	–	
February	0.97	*	(0.94–1.00)	
March	1.11	***	(1.08–1.14)	
April	1.05	**	(1.02–1.08)	
May	1.17	***	(1.14–1.20)	
June	1.23	***	(1.19–1.26)	
July	1.27	***	(1.24–1.31)	
August	1.35	***	(1.32–1.39)	
September	1.44	***	(1.41–1.48)	
October	1.39	***	(1.35–1.43)	
November	1.42	***	(1.38–1.46)	
December	1.44	***	(1.40–1.48)	
**Year**				
2010	1 (ref)	–	–	
2011	4.43		(0.59–33.32)	
2012	8.82	*	(1.18–66.14)	
2013	13.25	*	(1.77–99.41)	
2014	17.71	**	(2.36–132.84)	
2015	26.22	**	(3.50–196.61)	
2016	43.58	***	(5.81–326.87)	
*Total Observations (N)*	*1*,*227*,*238*			
*Total Groups (N)*	*33*,*179*			

IRR – Incidence Rate Ratios; 95% CI – 95% Confidence Intervals. Fixed effects Poisson regression, adjusted for continuous clinic covariates (number of health teams and number of appointments), year, and month. *p < 0.05; **p < 0.01; *** p < 0.001

### Appendix 9. Incidence rate ratios from a fixed effects poisson regression model examining the four-month lagged effects of neighbourhood violence on primary healthcare consultations for mental health

**Table:**

Characteristics	IRR		95% CI	
**Number of neighbourhood violent events (per month)**				
0	1 (ref)	–	–	
1	0.99		(0.97–1.01)	
2	1.04	**	(1.01–1.07)	
3	1.07	***	(1.03–1.11)	
4	1.05	*	(1.01–1.10)	
5+	0.93	**	(0.89–0.97)	
**Four-month lagged number of neighbourhood violent events (per month)**				
0	1 (ref)	–	–	
1	1.03	**	(1.01–1.05)	
2	1.05	***	(1.02–1.07)	
3	1.07	***	(1.03–1.11)	
4	1.06	**	(1.02–1.10)	
5+	0.95	*	(0.91–0.99)	
**Number of clinic health teams**	1.05	***	(1.03–1.06)	
**Number of clinic appointments**	1.00	***	(1.00–1.00)	
**Month**				
January	1 (ref)	–	–	
February	0.97	*	(0.94–1.00)	
March	1.11	***	(1.08–1.15)	
April	1.06	***	(1.03–1.09)	
May	1.18	***	(1.14–1.21)	
June	1.24	***	(1.21–1.28)	
July	1.29	***	(1.26–1.33)	
August	1.37	***	(1.33–1.41)	
September	1.46	***	(1.42–1.50)	
October	1.40	***	(1.36–1.43)	
November	1.43	***	(1.39–1.47)	
December	1.44	***	(1.40–1.48)	
**Year**				
2010	1 (ref)	–	–	
2011	4.25		(0.56–32.03)	
2012	8.26	*	(1.10–62.06)	
2013	12.55	*	(1.67–94.30)	
2014	16.53	**	(2.20–124.27)	
2015	24.44	**	(3.25–183.67)	
2016	40.68	***	(5.41–305.72)	
*Total Observations (N)*	*1*,*181*,*771*			
*Total Groups (N)*	*31*,*855*			

IRR – Incidence Rate Ratios; 95% CI – 95% Confidence Intervals. Fixed effects Poisson regression, adjusted for continuous clinic covariates (number of health teams and number of appointments), year, and month. *p < 0.05; **p < 0.01; *** p < 0.001

### Appendix 10. Incidence rate ratios from a fixed effects poisson regression model examining the five-month lagged effects of neighbourhood violence on primary healthcare consultations for mental health

**Table:**

Characteristics	IRR		95% CI	
**Number of neighbourhood violent events (per month)**				
0	1 (ref)	–	–	
1	1.00		(0.98–1.02)	
2	1.05	**	(1.02–1.07)	
3	1.08	***	(1.04–1.12)	
4	1.04		(1.00–1.09)	
5+	0.92	***	(0.88–0.96)	
**Five-month lagged number of neighbourhood violent events (per month)**				
0	1 (ref)	–	–	
1	1.02	*	(1.00–1.04)	
2	1.05	***	(1.02–1.07)	
3	1.05	*	(1.01–1.08)	
4	1.09	***	(1.04–1.13)	
5+	0.94	**	(0.90–0.98)	
**Number of clinic health teams**	1.05	***	(1.03–1.06)	
**Number of clinic appointments**	1.00	***	(1.00–1.00)	
**Month**				
January	1 (ref)	–	–	
February	0.98		(0.95–1.01)	
March	1.12	***	(1.09–1.15)	
April	1.07	***	(1.04–1.10)	
May	1.19	***	(1.15–1.22)	
June	1.25	***	(1.22–1.29)	
July	1.30	***	(1.27–1.34)	
August	1.39	***	(1.35–1.43)	
September	1.47	***	(1.43–1.51)	
October	1.40	***	(1.36–1.44)	
November	1.43	***	(1.39–1.47)	
December	1.45	***	(1.41–1.49)	
**Year**				
2010	1 (ref)	–	–	
2011	3.64		(0.48–27.49)	
2012	7.20		(0.96–54.21)	
2013	10.98	*	(1.46–82.68)	
2014	14.26	*	(1.89–107.41)	
2015	21.01	**	(2.79–158.27)	
2016	34.99	**	(4.65–263.58)	
*Total Observations (N)*	*1*,*139*,*890*			
*Total Groups (N)*	*30*,*733*			

IRR – Incidence Rate Ratios; 95% CI – 95% Confidence Intervals. Fixed effects Poisson regression, adjusted for continuous clinic covariates (number of health teams and number of appointments), year, and month. *p < 0.05; **p < 0.01; *** p < 0.001

### Appendix 11. Incidence rate ratios from a fixed effects poisson regression model examining the six-month lagged effects of neighbourhood violence on primary healthcare consultations for mental health

**Table:**

Characteristics	IRR		95% CI	
**Number of neighbourhood violent events (per month)**				
0	1 (ref)	–	–	
1	1.00		(0.98–1.02)	
2	1.05	***	(1.03–1.08)	
3	1.07	***	(1.04–1.11)	
4	1.05	*	(1.00–1.09)	
5+	0.93	**	(0.88–0.97)	
**Six-month lagged number of neighbourhood violent events (per month)**				
0	1 (ref)	–	–	
1	1.02	*	(1.00–1.04)	
2	1.03	*	(1.01–1.06)	
3	1.06	**	(1.02–1.09)	
4	1.05	*	(1.00–1.09)	
5+	0.91	***	(0.88–0.95)	
**Number of clinic health teams**	1.05	***	(1.03–1.06)	
**Number of clinic appointments**	1.00	***	(1.00–1.00)	
**Month**				
January	1 (ref)	–	–	
February	0.96	*	(0.93–0.99)	
March	1.12	***	(1.08–1.15)	
April	1.06	***	(1.03–1.09)	
May	1.19	***	(1.15–1.22)	
June	1.25	***	(1.21–1.28)	
July	1.30	***	(1.26–1.33)	
August	1.38	***	(1.34–1.42)	
September	1.47	***	(1.43–1.51)	
October	1.39	***	(1.35–1.43)	
November	1.43	***	(1.39–1.47)	
December	1.44	***	(1.40–1.48)	
**Year**				
2010	1 (ref)	–	–	
2011	3.06		(0.40–23.30)	
2012	5.97		(0.79–45.24)	
2013	9.30	*	(1.23–70.46)	
2014	11.89	*	(1.57–90.06)	
2015	17.54	**	(2.32–132.81)	
2016	29.24	**	(3.86–221.45)	
*Total Observations (N)*	*1*,*098*,*080*			
*Total Groups (N)*	*29*,*669*			

IRR – Incidence Rate Ratios; 95% CI – 95% Confidence Intervals. Fixed effects Poisson regression, adjusted for continuous clinic covariates (number of health teams and number of appointments), year, and month. *p < 0.05; **p < 0.01; *** p < 0.001

### Appendix 12. Incidence rate ratios from a fixed effects poisson regression model examining the effect of neighbourhood violence (any type) on primary healthcare consultations for mental health from interactions between violence and sex

**Table:**

Characteristics	IRR		95% CI	
**Number of neighbourhood violent events (per month)**				
0	1 (ref)	–	–	
1	1.02		(0.99–1.05)	
2	1.08	**	(1.03–1.13)	
3	1.10	**	(1.04–1.17)	
4	1.04		(0.97–1.11)	
5+	0.97		(0.90–1.04)	
**Number of neighbourhood violent events × Sex**				
0 × Female	1 (omitted)	–	–	
1 × Female	0.97		(0.94–1.01)	
2 × Female	0.96		(0.91–1.01)	
3 × Female	0.97		(0.90–1.04)	
4 × Female	0.99		(0.91–1.07)	
5+ × Female	0.94		(0.86–1.03)	
**Month**				
January	1 (ref)	–	–	
February	0.97	*	(0.94–1.00)	
March	1.09	***	(1.06–1.12)	
April	1.03	*	(1.00–1.06)	
May	1.15	***	(1.11–1.18)	
June	1.21	***	(1.18–1.24)	
July	1.27	***	(1.24–1.31)	
August	1.35	***	(1.31–1.39)	
September	1.45	***	(1.41–1.49)	
October	1.40	***	(1.36–1.43)	
November	1.43	***	(1.39–1.47)	
December	1.45	***	(1.41–1.49)	
**Year**				
2010	1 (ref)	–	–	
2011	4.82		(0.64–36.06)	
2012	10.29	*	(1.38–76.86)	
2013	14.46	**	(1.94–108.06)	
2014	20.31	**	(2.72–151.80)	
2015	29.79	**	(3.99–222.61)	
2016	48.88	***	(6.54–365.24)	
**Number of clinic health teams**	1.04	***	(1.03–1.06)	
**Number of clinic appointments**	1.00	***	(1.00–1.00)	
*Total Observations (N)*	*1*,*384*,*805*			
*Total Groups (N)*	*38*,*054*			

IRR – Incidence Rate Ratios; 95% CI – 95% Confidence Intervals. Fixed effects Poisson regression, adjusted for continuous clinic covariates (number of health teams and number of appointments), year, and month. *p < 0.05; **p < 0.01; *** p < 0.001. For interaction terms, the effect should be interpreted by multiplying the IRR of the main effect by the IRR of the interaction term

### Appendix 13. Incidence rate ratios from a fixed effects poisson regression model examining the effect of neighbourhood violence (any type) on primary healthcare consultations for mental health from interactions between violence and race/skin colour

**Table:**

Characteristics	IRR		95% CI	
**Number of neighbourhood violent events (per month)**				
0	1 (ref)	–	–	
1	1.02		(0.99–1.05)	
2	1.08	**	(1.03–1.13)	
3	1.10	**	(1.04–1.17)	
4	1.04		(0.97–1.11)	
5+	0.97		(0.90–1.04)	
**Number of neighbourhood violent events × Race/Skin Colour**				
0 × White	1 (ref)	–	–	
0 × Black	1 (omitted)	–	–	
0 × Pardo (mixed race)	1 (omitted)	–	–	
0 × Amarelo (Asian), Indigenous, Other or Unknown (Missing)	1 (omitted)	–	–	
1 × Black	0.98		(0.92–1.04)	
1 × Pardo (mixed race)	1.01		(0.97–1.05)	
1 × Amarelo (Asian), Indigenous, Other or Unknown (Missing)	0.92		(0.83–1.01)	
2 × Black	0.99		(0.91–1.08)	
2 × Pardo (mixed race)	1.01		(0.96–1.07)	
2 × Amarelo (Asian), Indigenous, Other or Unknown (Missing)	0.98		(0.86–1.11)	
3 × Black	1.04		(0.94–1.15)	
3 × Pardo (mixed race)	1.06		(0.98–1.14)	
3 × Amarelo (Asian), Indigenous, Other or Unknown (Missing)	0.68	**	(0.54–0.86)	
4 × Black	1.00		(0.88–1.13)	
4 × Pardo (mixed race)	1.02		(0.94–1.11)	
4 × Amarelo (Asian), Indigenous, Other or Unknown (Missing)	1.35	**	(1.13–1.63)	
5+ × Black	0.94		(0.82–1.07)	
5+ × Pardo (mixed race)	0.99		(0.90–1.09)	
5+ × Amarelo (Asian), Indigenous, Other or Unknown (Missing)	0.92		(0.74–1.14)	
**Month**				
January	1 (ref)	–	–	
February	0.97	*	(0.94–1.00)	
March	1.09	***	(1.06–1.12)	
April	1.03	*	(1.00–1.06)	
May	1.14	***	(1.11–1.18)	
June	1.21	***	(1.18–1.24)	
July	1.27	***	(1.24–1.31)	
August	1.35	***	(1.31–1.39)	
September	1.45	***	(1.41–1.49)	
October	1.40	***	(1.36–1.43)	
November	1.43	***	(1.39–1.46)	
December	1.45	***	(1.41–1.49)	
**Year**				
2010	1 (ref)	–	–	
2011	4.82		(0.64–36.07)	
2012	10.29	*	(1.38–76.89)	
2013	14.47	**	(1.94–108.13)	
2014	20.31	**	(2.72–151.80)	
2015	29.80	**	(3.99–222.68)	
2016	48.89	***	(6.54–365.28)	
**Number of clinic health teams**	1.05	***	(1.03–1.06)	
**Number of clinic appointments**	1.00	***	(1.00–1.00)	
*Total Observations (N)*	*1*,*384*,*805*			
*Total Groups (N)*	*38*,*054*			

IRR – Incidence Rate Ratios; 95% CI – 95% Confidence Intervals. Fixed effects Poisson regression, adjusted for continuous clinic covariates (number of health teams and number of appointments), year, and month. *p < 0.05; **p < 0.01; *** p < 0.001. For interaction terms, the effect should be interpreted by multiplying the IRR of the main effect by the IRR of the interaction term

### Appendix 14. Incidence rate ratios from a fixed effects poisson regression model examining the effect of neighbourhood violence (any type) on primary healthcare consultations for mental health from interactions between violence and education

**Table:**

Characteristics	IRR		95% CI	
**Number of neighbourhood violent events (per month)**				
0	1 (ref)	–	–	
1	1.01		(0.96–1.06)	
2	1.10	**	(1.03–1.18)	
3	1.12	*	(1.01–1.23)	
4	1.16	*	(1.03–1.30)	
5+	1.08		(0.96–1.22)	
**Number of neighbourhood violent events × Education**				
0 × None/pre-school/literacy class	1 (ref)	–	–	
0 × Elementary school	1 (omitted)	–	–	
0 × High school or higher education	1 (omitted)	–	–	
0 × None reported (missing)	1 (omitted)	–	–	
1 × Elementary school	1.02		(0.96–1.08)	
1 × High school or higher education	0.99		(0.92–1.05)	
1 × None reported (missing)	0.94		(0.88–1.01)	
2 × Elementary school	1.00		(0.92–1.08)	
2 × High school or higher education	0.99		(0.91–1.08)	
2 × None reported (missing)	0.83	***	(0.77–0.91)	
3 × Elementary school	1.03		(0.93–1.15)	
3 × High school or higher education	1.01		(0.90–1.14)	
3 × None reported (missing)	0.79	***	(0.70–0.89)	
4 × Elementary school	0.92		(0.81–1.05)	
4 × High school or higher education	0.95		(0.82–1.10)	
4 × None reported (missing)	0.78	***	(0.68–0.89)	
5+ × Elementary school	0.87		(0.76–1.00)	
5+ × High school or higher education	0.80	**	(0.68–0.94)	
5+ × None reported (missing)	0.80	**	(0.70–0.92)	
**Month**				
January	1 (ref)	–	–	
February	0.98		(0.95–1.00)	
March	1.10	***	(1.07–1.13)	
April	1.04	*	(1.01–1.07)	
May	1.15	***	(1.12–1.18)	
June	1.22	***	(1.19–1.26)	
July	1.28	***	(1.25–1.32)	
August	1.36	***	(1.32–1.40)	
September	1.46	***	(1.42–1.50)	
October	1.40	***	(1.37–1.44)	
November	1.44	***	(1.40–1.47)	
December	1.46	***	(1.42–1.50)	
**Year**				
2010	1 (ref)	–	–	
2011	4.84		(0.65–36.22)	
2012	10.29	*	(1.38–76.92)	
2013	14.49	**	(1.94–108.27)	
2014	20.38	**	(2.73–152.30)	
2015	29.90	**	(4.00–223.39)	
2016	49.08	***	(6.57–366.70)	
**Number of clinic health teams**	1.04	***	(1.03–1.06)	
**Number of clinic appointments**	1.00	***	(1.00–1.00)	
*Total Observations (N)*	*1*,*384*,*805*			
*Total Groups (N)*	*38*,*054*			

IRR – Incidence Rate Ratios; 95% CI – 95% Confidence Intervals. Fixed effects Poisson regression, adjusted for continuous clinic covariates (number of health teams and number of appointments), year, and month. *p < 0.05; **p < 0.01; ***p < 0.001. For interaction terms, the effect should be interpreted by multiplying the IRR of the main effect by the IRR of the interaction term

### Appendix 15. Incidence rate ratios from a fixed effects poisson regression model examining the effect of neighbourhood violence (any type) on primary healthcare consultations for mental health from interactions between violence and age group

**Table:**

Characteristics	IRR		95% CI	
**Number of neighbourhood violent events (per month)**				
0	1 (ref)	–	–	
1	0.90	*	(0.81–0.99)	
2	0.98		(0.86–1.12)	
3	0.90		(0.75–1.07)	
4	1.03		(0.86–1.23)	
5+	0.65	***	(0.52–0.82)	
**Number of neighbourhood violent events × Age Group**				
0 × 0–18	1 (ref)	–	–	
0 × 19–34 years	1 (omitted)	–	–	
0 × 35–54 years	1 (omitted)	–	–	
0 × 55–64 years	1 (omitted)	–	–	
0 × 65 + years	1 (omitted)	–	–	
1 × 19–34 years	1.16	*	(1.03–1.29)	
1 × 35–54 years	1.10		(0.99–1.22)	
1 × 55–64 years	1.14	*	(1.02–1.26)	
1 × 65 + years	1.11		(1.00–1.24)	
2 × 19–34 years	1.13		(0.97–1.32)	
2 × 35–54 years	1.05		(0.91–1.21)	
2 × 55–64 years	1.04		(0.90–1.20)	
2 × 65 + years	1.11		(0.96–1.28)	
3 × 19–34 years	1.27	*	(1.03–1.55)	
3 × 35–54 years	1.19		(0.99–1.43)	
3 × 55–64 years	1.26	*	(1.05–1.52)	
3 × 65 + years	1.16		(0.95–1.40)	
4 × 19–34 years	1.09		(0.88–1.35)	
4 × 35–54 years	0.96		(0.79–1.16)	
4 × 55–64 years	0.97		(0.80–1.18)	
4 × 65 + years	1.05		(0.86–1.28)	
5+ × 19–34 years	1.50	**	(1.16–1.95)	
5+ × 35–54 years	1.39	**	(1.10–1.76)	
5+ × 55–64 years	1.43	**	(1.12–1.82)	
5+ × 65 + years	1.48	**	(1.16–1.89)	
**Month**				
January	0.97	*	(0.94–1.00)	
February	1.09	***	(1.06–1.12)	
March	1.03	*	(1.00–1.06)	
April	1.14	***	(1.11–1.18)	
May	1.21	***	(1.18–1.24)	
June	1.27	***	(1.24–1.31)	
July	1.35	***	(1.31–1.39)	
August	1.45	***	(1.41–1.49)	
September	1.40	***	(1.36–1.43)	
October	1.43	***	(1.39–1.46)	
November	1.45	***	(1.41–1.49)	
December	0.97	*	(0.94–1.00)	
**Year**				
2010	4.82		(0.64–36.08)	
2011	10.29	*	(1.38–76.88)	
2012	14.46	**	(1.94–108.10)	
2013	20.31	**	(2.72–151.80)	
2014	29.80	**	(3.99–222.69)	
2015	48.90	***	(6.54–365.38)	
2016	4.82		(0.64–36.08)	
**Number of clinic health teams**	1.04	***	(1.03–1.06)	
**Number of clinic appointments**	1.00	***	(1.00–1.00)	
*Total Observations (N)*	*1*,*384*,*805*			
*Total Groups (N)*	*38*,*054*			

IRR – Incidence Rate Ratios; 95% CI – 95% Confidence Intervals. Fixed effects Poisson regression, adjusted for continuous clinic covariates (number of health teams and number of appointments), year, and month. *p < 0.05; **p < 0.01; *** p < 0.001. For interaction terms, the effect should be interpreted by multiplying the IRR of the main effect by the IRR of the interaction term

### Appendix 16. Incidence rate ratios from a fixed effects poisson regression model examining the effect of neighbourhood violence (any type) on primary healthcare consultations for mental health from interactions between violence and household income

**Table:**

Characteristics	IRR		95% CI	
**Number of neighbourhood violent events (per month)**				
0	1 (ref)	–	–	
1	1.01		(0.96–1.07)	
2	1.13	**	(1.05–1.21)	
3	1.15	**	(1.06–1.25)	
4	1.10		(0.99–1.22)	
5+	0.94		(0.83–1.06)	
**Number of neighbourhood violent events × Age Group**				
0 × Some salary reported to less than full minimum salary	1 (ref)	–	–	
0 × One minimum salary but less than two minimum salaries	1 (omitted)	–	–	
0 × Two minimum salaries or more	1 (omitted)	–	–	
0 × No salary reported (unknown or missing)	1 (omitted)	–	–	
1 × One minimum salary but less than two minimum salaries	0.98		(0.91–1.04)	
1 × Two minimum salaries or more	1.01		(0.92–1.12)	
1 × No salary reported (unknown or missing)	0.98		(0.93–1.04)	
2 × One minimum salary but less than two minimum salaries	0.96		(0.88–1.05)	
2 × Two minimum salaries or more	0.92		(0.80–1.05)	
2 × No salary reported (unknown or missing)	0.91	*	(0.84–0.98)	
3 × One minimum salary but less than two minimum salaries	1.01		(0.91–1.13)	
3 × Two minimum salaries or more	0.96		(0.82–1.13)	
3 × No salary reported (unknown or missing)	0.88	*	(0.80–0.97)	
4 × One minimum salary but less than two minimum salaries	0.97		(0.85–1.11)	
4 × Two minimum salaries or more	0.88		(0.72–1.08)	
4 × No salary reported (unknown or missing)	0.93		(0.82–1.04)	
5+ × One minimum salary but less than two minimum salaries	0.91		(0.77–1.07)	
5+ × Two minimum salaries or more	1.01		(0.81–1.27)	
5+ × No salary reported (unknown or missing)	1.02		(0.89–1.16)	
**Month**				
January	1 (ref)	–	–	
February	0.97	*	(0.94–1.00)	
March	1.09	***	(1.06–1.12)	
April	1.03	*	(1.00–1.06)	
May	1.15	***	(1.11–1.18)	
June	1.21	***	(1.18–1.25)	
July	1.27	***	(1.24–1.31)	
August	1.35	***	(1.31–1.39)	
September	1.45	***	(1.41–1.49)	
October	1.40	***	(1.36–1.43)	
November	1.43	***	(1.39–1.46)	
December	1.45	***	(1.41–1.49)	
**Year**				
2010	1 (ref)	–	–	
2011	4.82		(0.64–36.04)	
2012	10.29	*	(1.38–76.90)	
2013	14.46	**	(1.94–108.09)	
2014	20.33	**	(2.72–151.95)	
2015	29.79	**	(3.99–222.54)	
2016	48.81	***	(6.53–364.72)	
**Number of clinic health teams**	1.05	***	(1.03–1.06)	
**Number of clinic appointments**	1.00	***	(1.00–1.00)	
*Total Observations (N)*	*1*,*384*,*805*			
*Total Groups (N)*	*38*,*054*			

IRR – Incidence Rate Ratios; 95% CI – 95% Confidence Intervals. Fixed effects Poisson regression, adjusted for continuous clinic covariates (number of health teams and number of appointments), year, and month. *p < 0.05; **p < 0.01; *** p < 0.001. For interaction terms, the effect should be interpreted by multiplying the IRR of the main effect by the IRR of the interaction term

### Appendix 17. Incidence rate ratios from a fixed effects poisson regression model examining the effect of neighbourhood violence (any type) on primary healthcare consultations for mental health from interactions between violence and Bolsa Família-claiming family

**Table:**

Characteristics	IRR		95% CI	
**Number of neighbourhood violent events (per month)**				
0	1 (ref)	–	–	
1	1.00		(0.98–1.02)	
2	1.05	***	(1.03–1.08)	
3	1.08	***	(1.04–1.12)	
4	1.03		(0.99–1.08)	
5+	0.92	**	(0.88–0.97)	
**Number of neighbourhood violent events × Bolsa Família-claiming family**				
0 × No	1 (ref)	–	–	
0 × Yes	1 (omitted)	–	–	
1 × Yes	0.97		(0.91–1.03)	
2 × Yes	0.95		(0.88–1.03)	
3 × Yes	1.00		(0.90–1.11)	
4 × Yes	0.97		(0.85–1.10)	
5+ × Yes	1.04		(0.91–1.18)	
**Month**				
January	1 (ref)	–	–	
February	0.97	*	(0.94–1.00)	
March	1.09	***	(1.06–1.12)	
April	1.03	*	(1.00–1.06)	
May	1.14	***	(1.11–1.18)	
June	1.21	***	(1.18–1.24)	
July	1.27	***	(1.24–1.31)	
August	1.35	***	(1.31–1.39)	
September	1.45	***	(1.41–1.49)	
October	1.40	***	(1.36–1.43)	
November	1.43	***	(1.39–1.46)	
December	1.45	***	(1.41–1.49)	
**Year**				
2010	1 (ref)	–	–	
2011	4.82		(0.64–36.07)	
2012	10.29	*	(1.38–76.88)	
2013	14.46	**	(1.94–108.08)	
2014	20.31	**	(2.72–151.80)	
2015	29.80	**	(3.99–222.62)	
2016	48.88	***	(6.54–365.26)	
**Number of clinic health teams**	1.04	***	(1.03–1.06)	
**Number of clinic appointments**	1.00	***	(1.00–1.00)	
*Total Observations (N)*	*1*,*384*,*805*			
*Total Groups (N)*	*38*,*054*			

IRR – Incidence Rate Ratios; 95% CI – 95% Confidence Intervals. Fixed effects Poisson regression, adjusted for continuous clinic covariates (number of health teams and number of appointments), year, and month. *p < 0.05; **p < 0.01; *** p < 0.001. For interaction terms, the effect should be interpreted by multiplying the IRR of the main effect by the IRR of the interaction term

### Appendix 18. Incidence rate ratios from a fixed effects poisson regression model examining the effect of neighbourhood violence (any type) on primary healthcare consultations for mental health from interactions between violence and private healthcare insurance registration

**Table:**

Characteristics	IRR		95% CI	
**Number of neighbourhood violent events (per month)**				
0	1 (ref)	–	–	
1	1.00		(0.98–1.02)	
2	1.05	***	(1.02–1.08)	
3	1.08	***	(1.04–1.12)	
4	1.03		(0.99–1.07)	
5+	0.93	**	(0.89–0.97)	
**Number of neighbourhood violent events × Private healthcare insurance registration**				
0 × No	1 (ref)	–	–	
0 × Yes	1 (omitted)	–	–	
1 × Yes	0.97		(0.89–1.06)	
2 × Yes	0.92		(0.81–1.05)	
3 × Yes	0.98		(0.83–1.15)	
4 × Yes	0.90		(0.74–1.10)	
5+ × Yes	0.94		(0.75–1.19)	
**Month**				
January	1 (ref)	–	–	
February	0.97	*	(0.94–1.00)	
March	1.09	***	(1.06–1.12)	
April	1.03	*	(1.00–1.06)	
May	1.14	***	(1.11–1.18)	
June	1.21	***	(1.18–1.24)	
July	1.27	***	(1.24–1.31)	
August	1.35	***	(1.31–1.39)	
September	1.45	***	(1.41–1.49)	
October	1.40	***	(1.36–1.43)	
November	1.43	***	(1.39–1.47)	
December	1.45	***	(1.41–1.49)	
**Year**				
2010	1 (ref)	–	–	
2011	4.82		(0.64–36.07)	
2012	10.29	*	(1.38–76.87)	
2013	14.46	**	(1.94–108.08)	
2014	20.31	**	(2.72–151.80)	
2015	29.80	**	(3.99–222.62)	
2016	48.89	***	(6.54–365.31)	
**Number of clinic health teams**	1.04	***	(1.03–1.06)	
**Number of clinic appointments**	1.00	***	(1.00–1.00)	
*Total Observations (N)*	*1*,*384*,*805*			
*Total Groups (N)*	*38*,*054*			

IRR – Incidence Rate Ratios; 95% CI – 95% Confidence Intervals. Fixed effects Poisson regression, adjusted for continuous clinic covariates (number of health teams and number of appointments), year, and month. *p < 0.05; **p < 0.01; *** p < 0.001. For interaction terms, the effect should be interpreted by multiplying the IRR of the main effect by the IRR of the interaction term

### Appendix 19. Incidence rate ratios from a random effects poisson regression model examining the effect of (any type of violence categorised as 0–5 + violent events) on primary healthcare consultations for mental health, adjusted for socioeconomic characteristics

**Table:**

Characteristics	IRR		95% CI	
**Number of neighbourhood violent events (per month)**				
0	1 (ref)	–	–	
1	1.01		(0.99–1.03)	
2	1.05	***	(1.03–1.08)	
3	1.10	***	(1.06–1.13)	
4	1.04	*	(1.00–1.08)	
5+	0.93	**	(0.90–0.97)	
**Sex**				
Male	1 (ref)	–	–	
Female	0.85	***	(0.82–0.89)	
**Race/Skin Colour**				
White	1 (ref)	–	–	
Black	0.77	***	(0.73–0.81)	
Pardo (Mixed Race)	0.87	***	(0.83–0.90)	
Amarelo (Asian), Indigenous, Other or Unknown (Missing)	0.66	***	(0.60–0.73)	
**Education**				
None/pre-school/literacy class	1 (ref)	–	–	
Elementary school	0.98		(0.89–1.09)	
High school or higher education	0.73	***	(0.66–0.81)	
None reported (missing)	1.34	***	(1.23–1.47)	
**Age Group (years)**				
0–18	1 (ref)	–	–	
19–34	5.55	***	(5.12–6.02)	
35–54	13.45	***	(12.41–14.58)	
55–64	14.42	***	(13.26–15.68)	
65+	7.94	***	(7.31–8.63)	
**Household Income**				
Some salary reported to less than full minimum salary	1 (ref)	–	–	
More than one minimum salary but less than two minimum salaries	0.84	***	(0.79–0.89)	
Two minimum salaries or more	0.96		(0.88–1.04)	
No salary reported (unknown or missing)	0.83	***	(0.79–0.88)	
**Bolsa Família-claiming Family**				
No	1 (ref)	–	–	
Yes	0.91	**	(0.86–0.96)	
**Private Health Insurance Registration**				
No	1 (ref)	–	–	
Yes	0.73	***	(0.67–0.79)	
**Number of clinic health teams**	1.03	***	(1.02–1.04)	
**Number of clinic appointments**	1.00	***	(1.00–1.00)	
**Month**				
January	1 (ref)	–	–	
February	0.97	*	(0.94–1.00)	
March	1.10	***	(1.07–1.13)	
April	1.04	*	(1.01–1.07)	
May	1.16	***	(1.12–1.19)	
June	1.22	***	(1.19–1.26)	
July	1.29	***	(1.26–1.33)	
August	1.38	***	(1.34–1.42)	
September	1.48	***	(1.44–1.52)	
October	1.43	***	(1.39–1.47)	
November	1.47	***	(1.43–1.52)	
December	1.51	***	(1.47–1.56)	
**Year**				
2010	1 (ref)	–	–	
2011	6.02		(0.82–44.29)	
2012	12.80	*	(1.74–94.07)	
2013	17.98	**	(2.45–132.14)	
2014	25.38	**	(3.45–186.46)	
2015	37.59	***	(5.12–276.16)	
2016	63.65	***	(8.66–467.88)	
*Total Observations (N)*	*43*,*207*,*977*			
*Total Groups (N)*	*1*,*347*,*972*			

IRR – Incidence Rate Ratios; 95% CI – 95% Confidence Intervals. Random effects Poisson regression, adjusted for continuous clinic covariates (number of health teams and number of appointments), sex, race/skin colour, education level, age group, household income, private health insurance, Bolsa Família-receiving family, year, and month. *p < 0.05; **p < 0.01; *** p < 0.001

### Appendix 20. Incidence rate ratios from a fixed effects poisson regression model examining the effect of neighbourhood violence (any type of violence categorised as 0–10 + violent events) on primary healthcare consultations for mental health

**Table:**

Characteristics	IRR		95% CI	
**Number of neighbourhood violent events (per month)**				
0	1 (ref)	–	–	
1	1.00		(0.98–1.02)	
2	1.05	***	(1.02–1.07)	
3	1.08	***	(1.04–1.12)	
4	1.03		(0.99–1.07)	
5	0.95		(0.89–1.01)	
6	0.96		(0.90–1.02)	
7	0.87	**	(0.78–0.96)	
8	0.91		(0.82–1.00)	
9	0.68	***	(0.59–0.79)	
10+	0.99		(0.89–1.08)	
**Number of clinic health teams**	1.04	***	(1.03–1.06)	
**Number of clinic appointments**	1.00	***	(1.00–1.00)	
**Month**				
January	1 (ref)	–	–	
February	0.97	*	(0.94–1.00)	
March	1.09	***	(1.06–1.12)	
April	1.03	*	(1.00–1.06)	
May	1.15	***	(1.11–1.18)	
June	1.21	***	(1.18–1.24)	
July	1.27	***	(1.24–1.31)	
August	1.35	***	(1.31–1.39)	
September	1.45	***	(1.41–1.49)	
October	1.40	***	(1.36–1.44)	
November	1.43	***	(1.39–1.47)	
December	1.45	***	(1.41–1.49)	
**Year**				
2010	1 (ref)	–	–	
2011	4.82		(0.64–36.03)	
2012	10.28	*	(1.38–76.78)	
2013	14.46	**	(1.93–108.04)	
2014	20.35	**	(2.72–152.11)	
2015	29.79	**	(3.99–222.55)	
2016	48.87	***	(6.54–365.15)	
*Total Observations (N)*	*1*,*384*,*805*			
*Total Groups (N)*	*38*,*054*			

IRR – Incidence Rate Ratios; 95% CI – 95% Confidence Intervals. Fixed effects Poisson regression, adjusted for continuous clinic covariates (number of health teams and number of appointments), year, and month. *p < 0.05; **p < 0.01; *** p < 0.001. For interaction terms, the effect should be interpreted by multiplying the IRR of the main effect by the IRR of the interaction term

### Appendix 21. Incidence rate ratios from a weighted poisson regression model examining the effect of any type of violence (categorised as 0–5 + violent events) on primary healthcare consultations for mental health, weighted by inverse probability of treatment weights to adjust for individual-level confounding

**Table:**

Characteristics	IRR		95% CI	
**Number of neighbourhood violent events (per month)**				
0	1 (ref)	–	–	
1	1.06	**	(1.02–1.10)	
2	1.14	***	(1.09–1.20)	
3	1.21	***	(1.14–1.28)	
4	1.08	*	(1.02–1.15)	
5+	0.97		(0.91–1.04)	
**Sex**				
Male	1 (ref)	–	–	
Female	1.01		(0.95–1.06)	
**Race/Skin Colour**				
White	1 (ref)	–	–	
Black	0.77	***	(0.71–0.84)	
Pardo (Mixed Race)	0.87	***	(0.82–0.92)	
Amarelo (Asian), Indigenous, Other or Unknown (Missing)	0.66	***	(0.57–0.77)	
**Education**				
None/pre-school/literacy class	1 (ref)	–	–	
Elementary school	0.70	***	(0.64–0.78)	
High school or higher education	0.57	***	(0.51–0.64)	
None reported (missing)	1.14	*	(1.03–1.27)	
**Age Group (years)**				
0–18	1 (ref)	–	–	
19–34	4.78	***	(4.16–5.50)	
35–54	11.58	***	(10.17–13.17)	
55–64	13.05	***	(11.45–14.87)	
65+	7.55	***	(6.61–8.62)	
**Household Income**				
Some salary reported to less than full minimum salary	1 (ref)	–	–	
More than one minimum salary but less than two minimum salaries	0.87	**	(0.79–0.95)	
Two minimum salaries or more	1.08		(0.93–1.25)	
No salary reported (unknown or missing)	0.74	***	(0.68–0.81)	
**Bolsa Família-claiming Family**				
No	1 (ref)	–	–	
Yes	0.93		(0.86–1.01)	
**Private Health Insurance Registration**				
No	1 (ref)	–	–	
Yes	0.68	***	(0.61–0.76)	
**Number of clinic health teams**				
**Number of clinic appointments**				
**Month**				
January	1 (ref)	–	–	
February	0.96		(0.92–1.01)	
March	1.12	***	(1.06–1.17)	
April	1.03		(0.98–1.08)	
May	1.17	***	(1.11–1.22)	
June	1.22	***	(1.16–1.28)	
July	1.33	***	(1.27–1.40)	
August	1.36	***	(1.30–1.43)	
September	1.51	***	(1.44–1.58)	
October	1.47	***	(1.40–1.54)	
November	1.53	***	(1.46–1.60)	
December	1.64	***	(1.57–1.72)	
**Year**				
2010	1 (ref)	–	–	
2011	9.36	*	(1.31–66.93)	
2012	20.07	**	(2.82–142.81)	
2013	30.01	**	(4.22–213.36)	
2014	40.59	***	(5.71–288.48)	
2015	60.73	***	(8.55–431.50)	
2016	111.15	***	(15.65–789.67)	
*Total Observations (N)*	*43*,*207*,*977*			
*Total Groups (N)*	*1*,*347*,*972*			

IRR – Incidence Rate Ratios; 95% CI – 95% Confidence Intervals. Weighted Poisson regression using inverse probability of treatment weights (IPTW), adjusted for, sex, race/skin colour, education level, age group, household income, private health insurance, Bolsa Família-receiving family, and continuous clinic covariates (number of health teams and number of appointments), year, and month. Robust standard errors clustered at the individual level. *p < 0.05; **p < 0.01; ***p < 0.001

### Appendix 22. Incidence rate ratios from a weighted random-effects poisson regression model examining the effect of any type of violence (categorised as 0–5 + violent events) on primary healthcare consultations for mental health, weighted by inverse probability of treatment weights to adjust for individual-level confounding

**Table:**

Characteristics	IRR		95% CI	
**Number of neighbourhood violent events (per month)**				
0	1 (ref)	–	–	
1	1.01		(0.99–1.03)	
2	1.05	***	(1.03–1.08)	
3	1.10	***	(1.06–1.13)	
4	1.04	*	(1.00–1.08)	
5+	0.93	**	(0.90–0.97)	
**Sex**				
Male	1 (ref)	–	–	
Female	0.85	***	(0.82–0.89)	
**Race/Skin Colour**				
White	1 (ref)	–	–	
Black	0.77	***	(0.73–0.81)	
Pardo (Mixed Race)	0.87	***	(0.83–0.90)	
Amarelo (Asian), Indigenous, Other or Unknown (Missing)	0.66	***	(0.60–0.73)	
**Education**				
None/pre-school/literacy class	1 (ref)	–	–	
Elementary school	0.98		(0.89–1.09)	
High school or higher education	0.73	***	(0.66–0.81)	
None reported (missing)	1.34	***	(1.23–1.47)	
**Age Group (years)**				
0–18	1 (ref)	–	–	
19–34	5.55	***	(5.12–6.02)	
35–54	13.45	***	(12.41–14.58)	
55–64	14.42	***	(13.26–15.68)	
65+	7.94	***	(7.31–8.63)	
**Household Income**				
Some salary reported to less than full minimum salary	1 (ref)	–	–	
More than one minimum salary but less than two minimum salaries	0.84	***	(0.79–0.89)	
Two minimum salaries or more	0.96		(0.88–1.04)	
No salary reported (unknown or missing)	0.83	***	(0.79–0.88)	
**Bolsa Família-claiming Family**				
No	1 (ref)	–	–	
Yes	0.91	**	(0.86–0.96)	
**Private Health Insurance Registration**				
No	1 (ref)	–	–	
Yes	0.73	***	(0.67–0.79)	
**Number of clinic health teams**	1.03	***	(1.02–1.04)	
**Number of clinic appointments**	1.00	***	(1.00–1.00)	
**Month**				
January	1 (ref)	–	–	
February	0.97	*	(0.94–1.00)	
March	1.10	***	(1.07–1.13)	
April	1.04	*	(1.01–1.07)	
May	1.16	***	(1.12–1.19)	
June	1.22	***	(1.19–1.26)	
July	1.29	***	(1.26–1.33)	
August	1.38	***	(1.34–1.42)	
September	1.48	***	(1.44–1.52)	
October	1.43	***	(1.39–1.47)	
November	1.47	***	(1.43–1.52)	
December	1.51	***	(1.47–1.56)	
**Year**				
2010	1 (ref)	–	–	
2011	6.02		(0.82–44.29)	
2012	12.80	*	(1.74–94.07)	
2013	17.98	**	(2.45–132.14)	
2014	25.38	**	(3.45–186.46)	
2015	37.59	***	(5.12–276.16)	
2016	63.65	***	(8.66–467.88)	
*Total Observations (N)*	*43*,*207*,*977*			
*Total Groups (N)*	*1*,*347*,*972*			

IRR – Incidence Rate Ratios; 95% CI – 95% Confidence Intervals. Random-effects Poisson regression model using inverse probability of treatment weights (IPTW), adjusted for sex, race/skin colour, education level, age group, household income, private health insurance, Bolsa Família-receiving family, and continuous clinic covariates (number of health teams and number of appointments), year, and month. Robust standard errors clustered at the individual level. **p* < 0.05; ***p* < 0.01; ****p* < 0.001
